# Investigation of Heart Rate Variability Indices in Motion Sickness

**DOI:** 10.3390/s26072114

**Published:** 2026-03-28

**Authors:** Alfonso Maria Ponsiglione, Lorena Guerrini, Simona Pierucci, Vittorio Santoriello, Maria Romano, Marco Recenti, Hannes Petersen, Paolo Gargiulo, Carlo Ricciardi

**Affiliations:** 1 Department of Electrical Engineering and Information Technology, University of Naples Federico II, 80138 Naples, Italy; alfonsomaria.ponsiglione@unina.it (A.M.P.); maria.romano@unina.it (M.R.); carlo.ricciardi@unina.it (C.R.); 2 Institute of Biomedical and Neural Engineering, Reykjavik University, 102 Reykjavík, Iceland; lorena22@ru.is (L.G.); pieruccisimona@hotmail.it (S.P.); marcor@ru.is (M.R.); paolo@ru.is (P.G.); 3 Department of Anatomy, School of Medicine, University of Iceland, 102 Reykjavík, Iceland; hpet@ru.is; 4 Department of Science, Landspitali University Hospital, 105 Reykjavik, Iceland

**Keywords:** heart rate variability, motion sickness, biosignals, autonomic nervous system, virtual reality, kinetosis

## Abstract

Motion sickness (MS), or kinetosis, is a condition experienced by some individuals in response to rhythmic or irregular body motion. Multiple studies have explored its neurobiological mechanisms and countermeasures, with the sensory-conflict hypothesis remaining the most accepted explanation. Heart-rate variability (HRV) and electrocardiography provide complementary autonomic nervous system perspectives that may support MS assessments. From an applied viewpoint, reliable HRV markers could enable the early detection and continuous monitoring of MS in real-world contexts, such as autonomous vehicles, where passenger comfort and safety are critical, motivating contact-free cardiac sensing for unobtrusive monitoring. This systematic review examines the value of HRV indices in MS, conducted under PRISMA guidelines across PubMed, Scopus, and the Web of Science. The included studies were grouped into four categories based on the methods used to induce MS: mechanical stimulus, real trip, visual stimulus, and virtual reality. Aggregated findings indicate that frequency–domain metrics, particularly the low frequency (LF)/high frequency (HF) ratio, HF power, and mean heart rate (mHR), are most frequently reported in relation to MS. Overall, autonomic dysregulation likely contributes to MS susceptibility, but standardized protocols are needed to validate HRV as a reliable marker.

## 1. Introduction

Motion sickness (MS), also known as kinetosis, is the state of being dizzy or nauseated because of motion [1]. Its underlying mechanisms are not yet fully understood, although the most widely accepted explanation is the “sensory conflict and neural mismatch” theory, which is currently the most accepted [2,3]. According to this model, MS arises when there is a mismatch between actual sensory inputs, e.g., vestibular (inner ear), proprioceptive, and visual signals, and the expected sensory patterns generated by the brain, versus expected sensory input [4,5]. Neurotransmitters, including acetylcholine and histamine, are implicated in these processes, which explains the effectiveness of anticholinergic and antihistamine medications as treatments [6,7,8].

Although often associated with nausea and vomiting, MS encompasses a much broader range of symptoms, including dizziness, cold sweating, pallor, and cognitive effects, making it a multifaceted syndrome rather than a single symptom disorder [9]. Recent consensus guidelines, such as those developed by the Classification Committee of the Bárány Society, have proposed standardized diagnostic criteria for MS to improve research comparability and clinical assessment [10].

There are many different types of MS. The main subtypes include the following:

- classic MS as carsickness or seasickness [11,12];
- Visually Induced Motion Sickness (VIMS), when these symptoms are triggered by visual stimulation without actual physical motion [13,14];
- space motion sickness (SMS) [12,15], a common and significant challenge for astronauts;
- virtual reality (VR)-induced MS (including cybersickness) arises during immersion in VR environments and is caused by the conflict between visual and vestibular input, with the peculiarity that, in most cases, the body is completely still while the perceived world is in motion [11,16,17].

Research on VIMS has shown that visual–vestibular discrepancies play a key role in triggering symptoms, highlighting the importance of the visual modality in both real and simulated motion environments. Within this context, VR is increasingly being recognized as a valuable tool for replicating the feeling of MS symptoms in certain individuals [7,18,19]. During VR-driven experimental protocols, the collection of physiological signals from subjects enables the study of physiological responses related to MS. Recenti et al. (2021) conducted an innovative VR experiment to induce MS and adopted biosignal features to classify the subjects’ conditions through machine learning (ML) algorithms [18].

**Figure 1:**
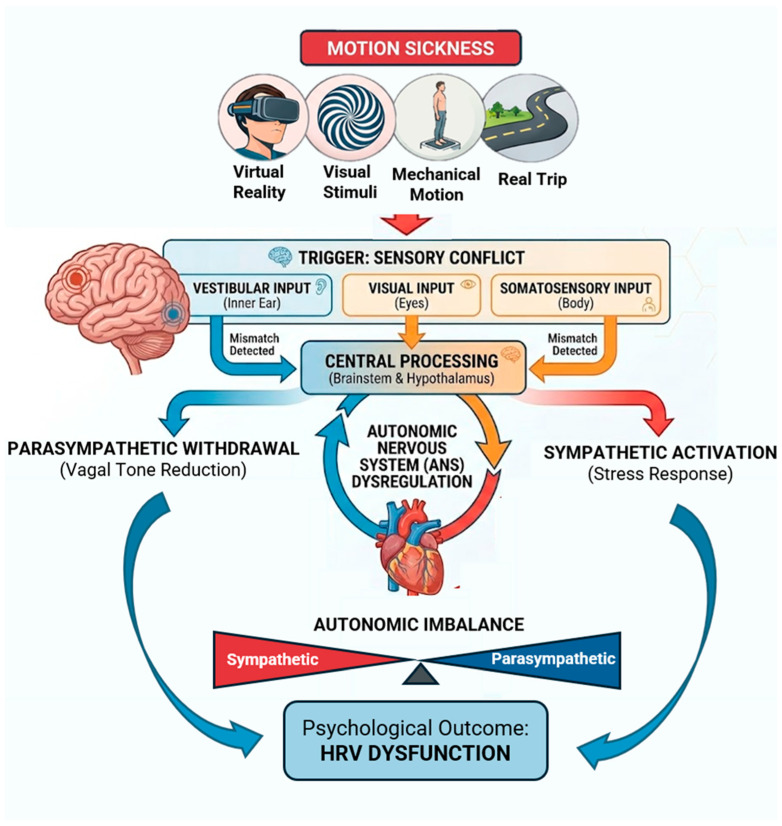
Relationship among motion sickness mechanisms, autonomic pathways, and HRV markers.

The relevance of MS is also evident from epidemiological data: up to 25% of the travelers experience MS within 3 days from the start of a ship travel [6]. The incidence of MS during 226 cruise trips was 4.2 per 1000 person per day, while MS is 0.13% of individuals who ride on trains, between 10% and 31% of student aviators, 80% of astronauts (space sickness), and up to 4% for subjects driving rally cars and sitting in the back seats or reading during a trip (car sickness) [6,20,21]. Moreover, females are more susceptible to MS than males with regard to the rate and grade of symptoms, in particular during menstruation [22,23]. By contrast, MS is rare in children under two years, while children 6–12 years old have greater susceptibility, which declines through adolescence. Finally, MS is less frequent in adults and rarely occurs after the age of 50 years [24]. Nevertheless, when it does occur, it provokes significant changes in the Autonomic Nervous System (ANS) activity [25,26] (e.g., cardiovascular, sudomotor, gastrointestinal responses). In this context, heart rate variability (HRV) provides a non-invasive window into cardiac autonomic modulation, reflecting the activity of the sympathetic and vagal components of the ANS on the sinus node of the heart [27]. HRV indicates the fluctuations in the time intervals between adjacent heartbeats and reflects the ability to adjust the heart rate (HR) in response to varying situations and internal and external stimuli. Its analysis enables an evaluation of overall cardiac health and autonomic modulation of the HR, which is accountable for regulating cardiac activity [21,25,26,28,29,30,31,32]. In fact, HRV has long been recognized as a reliable non-invasive marker of autonomic function [33], with reductions in variability often associated with pathological conditions or impaired regulatory capacity. HRV represents the dynamic balance between sympathetic and parasympathetic influences on the heart, and its measurement provides insights into both short-term adaptability and long-term health outcomes [6]. Moreover, contemporary research emphasises how HRV is influenced by psychological, as well as physiological, factors, suggesting its relevance as a biomarker not only for cardiovascular health but also for stress, emotional regulation, postural control tasks, and pain [34,35,36,37]. HRV is not specific to MS per se; rather, it is a non-invasive marker of cardiac autonomic modulation, which is affected by MS. Human body physiology, including cardiac activity, is influenced by the ANS, which consists of two subdivisions: the sympathetic and parasympathetic nervous systems. The sympathetic system is activated during stressful conditions, such as fear or the fight-or-flight response, eventually resulting in increased HR and sweat production. On the other hand, the parasympathetic system controls rest and digestion, leading to a decrease in the HR and, together with the action of the sympathetic nerve, contributes to the maintenance of homeostasis [38]. A representation of the relationship among MS, HRV, and ANS is illustrated in Figure 1.

Physiological factors such as age and gender influence HRV [39], as well as lifestyle factors [40,41,42,43]. Additionally, HRV tends to decrease during stressful situations due to the reduced parasympathetic activation [34,44]; physically healthy individuals with untreated major depressive disorder, whether with or without comorbid anxiety, have been found to have a reduced HR [45,46,47,48]. An HRV assessment is usually performed using different methods: linear time-based methods, non-linear methods, and frequency-based methods [33,49,50]. Time-domain, as well as frequency-domain, parameters can be used to quantify the HRV; the former are statistical measurements extracted from the interbeat interval time series, while the latter are used to calculate the absolute or relative signal power within the very-low-frequency (VLF) band, below 0.04 Hz, low-frequency (LF) band, ranging from 0.04 to 0.15 Hz, and high-frequency (HF) band, ranging from 0.15 to 0.4 Hz [33]. Furthermore, non-linear HRV parameters aim at quantifying the unpredictability, irregularity, and complexity of the interbeat intervals [51]. The physiological HRV control mechanism responsible for MS responses is still under investigation, and the most meaningful HRV parameters that are involved in MS are not well-known.

In this context, this study aims to conduct a systematic review of the literature to investigate the state of the art regarding the relationships between MS and HRV indices, by highlighting evidence from those studies focusing on the variation in HRV parameters influenced by MS, providing a detailed analysis and investigation of multiple HRV indices, beyond the measurement of the average HR.

The relationship between HRV and MS could be of interest from many perspectives. From an applied standpoint, identifying reliable HRV markers associated with the early phases of MS could support the early detection and monitoring of MS in real-world contexts (e.g., the use of autonomous vehicles [52]), given the importance of HRV as a non-invasive marker of cardiac autonomic modulation. Moreover, the study of this relationship may foster the development of innovative, non-contact methods for assessing cardiac activity, enabling unobtrusive monitoring in naturalistic environments. Beyond these practical implications, examining HRV in the context of MS could also contribute to a deeper understanding of the autonomic mechanisms underlying susceptibility and adaptation to motion-related discomfort, thus offering new insights for both clinical applications and research. Despite its potential relevance, the available evidence remains fragmented, with considerable heterogeneity across studies in terms of experimental paradigms, recording durations, and HRV measures. As a result, the current state of the literature is still difficult to interpret in a unified way, highlighting the need for a focused and systematic synthesis. In this context, the present review aims not only to summarise the existing literature but also to critically examine the HRV indices investigated in relation to MS, identify methodological limitations that affect comparability across studies, and highlight key gaps and future directions for research.

## 2. Materials and Methods

### 2.1. Information Sources and Search Strategy

This systematic review was performed according to the Preferred Reporting Items for Systematic Reviews and Meta-Analyses (PRISMA) guidelines [53,54]. The research has been conducted using three databases: Scopus, PubMed, and Web of Science. The adopted query ((“Heart rate variability” OR “HRV”) AND “Motion sickness”) has been the query performed to search in both the “Title” and “Abstract” for all the databases.

### 2.2. Eligibility Criteria and Selection Process

The literature screening was therefore carried out considering these inclusion criteria:

- Focus on the study of relationships between HRV indices and MS.
- Clear description of the experimental protocol used to study MS.
- Explicit discussion and details on HRV parameters’ changes in the presence of MS.

**Figure 2:**
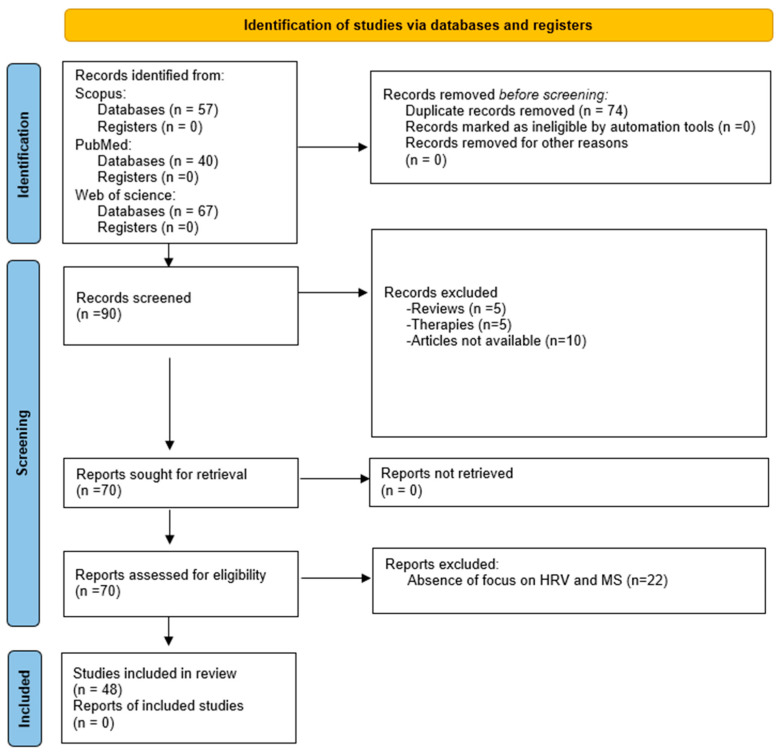
Workflow for the screening of the articles.

It is worth highlighting that we excluded studies on MS that, despite measuring physiological signals including ECG and reporting some parameters (e.g., mean HR), do not provide an in-depth discussion on HRV parameters or an explicit focus/objective on the study of HRV. In addition, non-English articles, duplicates, reviews, and papers not available in the first phase of research were excluded. Figure 2 represents the workflow of the review according to the PRISMA flowchart.

Papers published from 1998 (the year of the first paper published on this topic) to March 2025 were included in the search. Overall, 164 papers were found in the three databases, of which 90 were included after the removal of duplicates; 10 were excluded from the screening of the title and abstract because they were systematic reviews or focused on therapies, while 10 documents were excluded because the full text was not available. Of 70 articles, 22 were excluded due to the absence of a focus on HRV and MS. Finally, 48 papers were included in this systematic review, reflecting the scarcity of robust evidence in the diagnosis of motion sickness.

### 2.3. Data Collection and Clustering

First, data collection was conducted to gather the basic information regarding the documents to be screened (i.e., authors, title, year of publication, DOI, and abstract). The search was conducted in March 2025 and covered the literature from the earliest publication on the topic through March 2025. Two independent reviewers screened the documents for inclusion/exclusion criteria, following the PRISMA flowchart: first removing duplicates, then checking the title and abstract before moving to a deeper investigation with full-text reading and data clustering. No automation tools were used during the screening process. In the event of disagreement between the two independent reviewers, a third reviewer selected from among the authors would have been consulted. However, no disagreements occurred. The included studies were grouped and discussed as follows.

### 2.4. HRV Indices

The studies included in this review used various HRV indices, including time, frequency, and nonlinear domain indices [33,55,56].

In the time-domain, metrics included the mean interbeat interval (mRR, MeanNN, IBI, RR-I), the standard deviation of NN intervals (SDNN/SDRR), the root mean square of successive differences (RMSSD), the standard deviation of successive differences (SDSD), the percentage of adjacent intervals differing by more than 20 ms or 50 ms (pNN20, pNN50), the coefficient of variation of RR intervals (CVRR) (to quantify overall or short-term variability in interbeat intervals, reflecting vagal and total autonomic modulation [57,58]), mean absolute deviation (MAD), HR standard deviation (HR_std) and average HR, and the Stress Index (SI).

SDNN and SDRR were considered global indices of overall variability reflecting combined sympathetic and parasympathetic influences, whereas RMSSD, SDSD, pNN20, and pNN50 were interpreted as markers predominantly reflecting short-term vagally mediated cardiac modulation.

Other features include SD1 and SD2, which are derived from the Poincaré plot and describe short-term and long-term variability in the signal, respectively. SD1 reflects beat-to-beat or rapid fluctuations, while SD2 captures slower, more global changes over time.

In addition, Sample Entropy and Fuzzy Entropy are nonlinear measures used to quantify the irregularity and complexity of the signal. Higher values generally indicate greater unpredictability, with Fuzzy Entropy often considered more robust than Sample Entropy when analyzing short or noisy physiological signals [59]. Frequency–domain parameters were derived from a power spectral density analysis and included VLF, LF, HF, total power (PTOT), and their logarithmic transformations (lnHF, lnVLF, ln (LF/HF)). Other features included normalized units (nLF, nHF), proportional indices (HF/(LF + HF), LF/(LF + HF), HF/(LF + HF) × 100), and band ratios (LF/HF, VLF/HF, lnVLF/lnHF). HF power (0.15–0.40 Hz) was interpreted as an index of respiratory-linked vagal modulation, whereas LF power (0.04–0.15 Hz) was cautiously interpreted as reflecting baroreflex-mediated modulation, commonly interpreted as markers of sympathetic–parasympathetic balance [60]. The LF component is modulated by both the sympathetic and parasympathetic nervous systems [27], consistent with contemporary evidence challenging its use as a pure sympathetic marker. The authors in the study [61] assert that LF power seems to provide an index not of cardiac sympathetic tone but of baroreflex function. Regarding the LF/HF ratio, classically considered as the sympatho-vagal balance, a study [62] affirmed that LF/HF data cannot accurately quantify cardiac “sympatho vagal balance” either in health or disease.

VLF was considered to reflect slower regulatory and neuroendocrine influences, though its physiological interpretation remains less well established in short-term recordings.

## 3. Results

### 3.1. Research Trend and Document Types

**Figure 3:**
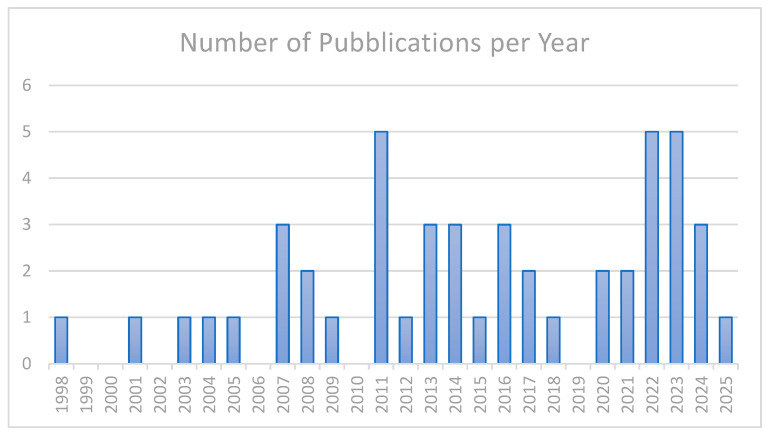
Temporal trend of publications on HRV and MS.

The articles published on the topics have gradually increased over the years, as shown in Figure 3. A total of 48 articles were included in the results of the systematic review, from 1998 to 2025. It is possible to see an increase in publications in the last decade with respect to the beginning of 2000. These articles were carefully selected based on specific criteria and their contribution to the topic of interest. Of the included articles, 85% were published in journals rather than conferences, confirming the growing interest in this topic.

**Figure 4:**
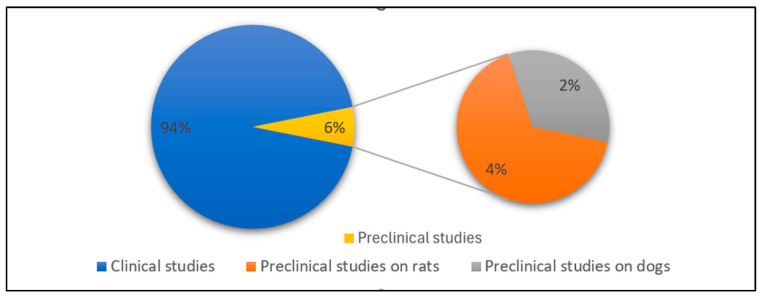
Distribution of studies according to the type of study: clinical vs. preclinical studies.

Furthermore, as shown in Figure 4, among the included studies, 45 out of 48 were conducted on humans, while 3 were preclinical studies [63,64,65].

#### Population Characteristics

The sample size of the reviewed studies varied considerably, ranging from 5 to 119 participants, while the mean age of the subjects included was in a quite narrow range because all the studies included subjects aged between 18 and 42 years. However, the majority of studies focused on samples with a mean age between 20 and 30 years. The distribution according to gender was unbalanced, and, surprisingly, males (50%) were studied more than females (17%), with the remaining percentage related to missing information. This finding contrasts the epidemiology of the pathology [1,22], which indicates that females are more susceptible to MS than males. Moreover, all the reviewed studies focused exclusively on healthy subjects, and no pathological conditions were investigated.

### 3.2. Experimental Approaches Adopted to Study MS

**Figure 5:**
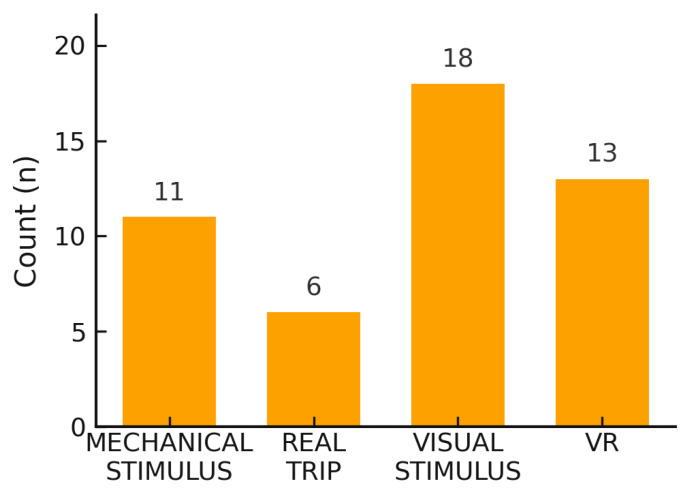
Methods for the induction of MS in subjects undergoing the experiments.

The included articles were grouped into four categories according to the methods used to induce MS: mechanical stimulus, real trip, visual stimulus, and VR. As illustrated in Figure 5, visual stimuli were the most frequently used, followed by mechanical and VR stimuli, while real travel experiences were the least frequently investigated among the included studies. In the next paragraphs, the results are analyzed according to these four methods of MS induction, extracting information on the number of subjects involved, mean age, time- and frequency-domain features, the device used for signal acquisition, the window over which HRV was calculated, the sampling rate (SR), the proposed signal-processing algorithm, and the main results regarding the relationship between HRV and MS, including the HRV features associated with MS.

#### 3.2.1. Visual Stimuli to Study MS

Table 1 shows the studies employing visual stimuli to study MS.

Of the 48 studies included in this review, 18 induced MS through visual stimuli, analyzing the relationships between HRV parameters, both in the time and frequency domains, and VIMS, as shown in Table 1. Among the HRV parameters examined in the frequency domain, most studies focus on the components of the power spectrum, specifically VLF, LF, and HF, as well as their normalized versions, and other parameters related to the HF component. Only one study specifically investigates respiratory sinus arrhythmia (RSA) [81]. In studies analyzing HRV parameters in the time domain, the mHR, the mean of the RR series (mRR), and RR intervals between consecutive beats (RR-I) are assessed; in some cases, the SDNN, pNN50, and the RMSSD are also considered. mHR was the most used in this context. All the studies that induce MS through visual stimuli report correlations between HRV changes and the onset of MS. These results were obtained using different sickness-induction techniques, ranging from the use of an optokinetic drum to the projection of highly stimulating videos, both in 2D and 3D, on screens of various sizes. A 3D stimulus has been employed in four papers [66,67,70,71], while the other employed generic visual stimuli with different patterns. Moreover, the sample size was between 14 and 59 subjects, while the mean age ranged between 20 and 30 years old. The balance between the sympathetic and parasympathetic nervous systems is the focus of the studies by Naqvi et al. [66] and Park et al. [67], who conducted comparative experiments between watching a video in 2D and in 3D. In 2013, Naqvi et al. [66] aimed to identify differences in VIMS levels using a 2D and a 3D stimulus by including 39 healthy subjects. The experimental procedure included three tasks: closing the eyes for 5 min, opening the eyes for 5 min, and watching the film for 10 min. An increased LF/HF ratio was found with the 2D video compared to the 3D, while subjects exposed to 3D images reported more subjective symptoms of VIMS. Park et al. [67] focused on comparing the effects of 2D and 3D on visual fatigue, cognitive functions, and autonomic measures in 30 healthy participants (15 in the 2D group, 15 in the 3D group) with a mean age of 23 years, and on testing the hypothesis that no significant differences would exist between the two groups. Park et al.’s study proposed the use of the VLF/HF ratio and HRV as physiological indicators of the visual cognitive load induced by 3D technology, integrating concepts of the heart–brain connection. The study revealed an increased VLF/HF ratio in the 3D group, along with an increase in SDNN. The monitoring and prediction of VIMS are the main focus of the studies by Tanaka et al. [68] and Keshavarz et al. [69] in 2022. Tanaka et al. [68] focused on the levels of VIMS and aimed at developing a technique for the estimation of continuous changes in the degree of VIMS by using physiological indices, i.e., HRV, showing video images to 41 healthy subjects (mean age of 27 years). In this paper, a multiple regression model based on physiological indices was implemented and was able to estimate the degree of VIMS better than a subjective score. By contrast, the study by Keshavarz et al. [69] aimed to determine whether ML techniques, in combination with physiological measures, could be used to detect and predict the severity of VIMS symptoms in real time. A total of 43 healthy subjects, 25 of whom were female, were exposed to a 15 min video designed to induce VIMS. Symptom severity was assessed using the Fast Motion Sickness Scale (FMS) and before and after the video using the SSQ. In addition, ECG and EGG signals, electrodermal activity (EDA), body and facial temperature, and respiration were analyzed. The study revealed that cardiovascular measures yielded inconclusive results for detecting VIMS: HR and HRV were only moderately correlated with VIMS, but they proved to be relevant parameters for ML analysis, suggesting that an increase in HR may represent a condition of psychological stress during VIMS.

Differently, Wibirama et al. (2014) used the SSQ, ECG, and 3D gaze tracking to investigate VIMS in a cohort of 40 healthy subjects (mean age = 22.5 years) [70]. ECG showed that horizontal motion and vertical motion were contributing factors to VIMS. In a subsequent study conducted on the same cohort, Wibirama et al. (2018) filled the gap in the literature among SSQ, depth gaze behavior, and HRV in a stereoscopic 3D motion picture [71]. The aim was to examine whether fixing the gaze on a single point can reduce VIMS symptoms. The authors highlight an increase in the LF/HF ratio and fluctuating HF values during the highly stimulating video and suggest that gaze fixation may represent a strategy to mitigate MS. Indeed, a decrease in LF/HF values and a stabilization of HF were observed.

The cardiovagal response to MS was investigated by LaCount et al. (2009) [72] and in 2011 [73] using a visual display of stripes projected on a screen that covered the entire visual field, while the subjects (19 with a mean age of 29.1 years in the first study, and 17 with a mean age of 28.4 years in the second, all women prone to MS) laid in a supine position inside an MRI scanner for up to 20 min. In the 2009 study, HF decreased during the onset of nausea and increased before the development of strong nausea. While the 2009 study focused exclusively on the dynamic cardiovagal response, the 2011 study was broader, also analyzing the skin conductance response (SCR), respiratory rate, and subjective ratings of nausea. Both studies confirmed a marked increase in the HR and SCR with nausea, along with a reduction in the vagal tone (HF) and in dynamic HF peaks before transitions to higher levels of nausea.

The studies by Kim et al. [74], Sclocco et al. [75], and Molefi et al. [76] also conclude that VIMS is associated with reduced parasympathetic activity and increased sympathetic activity, albeit with different methodological approaches. In all three cases, MS was induced by the presentation of moving black-and-white horizontal stripes; in the studies by Kim and Sclocco, the visual stimuli were delivered while participants were inside a functional magnetic resonance imaging (fMRI) scanner, whereas the study by Molefi did not involve fMRI.

The aim of Kim et al. was to evaluate the brain circuits of the ANS in response to nausea by combining fMRI with an algorithm for estimating HF power. The purpose of Sclocco et al. [75] was to examine sympathetic and cardiovagal modulation through the skin conductance level (SCL), HR, and HF power, to evaluate autonomic responses during nauseogenic visual stimulation used to induce vection in 25 susceptible females. Molefi’s study differed in its exclusive use of ECG signals and the adoption of a nonlinear HRV analysis; the goal was to assess complex metrics (entropy, chaos, and indices of sympathetic and vagal control) to explore autonomic changes during the onset of nausea. All three studies involved healthy subjects highly susceptible to MS: Kim and Sclocco included only female participants (21 with a mean age of 28.4 years, and 17 with a mean age between 24 and 27 years, respectively), while Molefi included 14 participants, 12 of whom were female. In addition, Sclocco included a control group of eight subjects not susceptible to nausea. In the study by Kim et al. (2011) [74], two brain areas were found to be positively correlated with HF-HRV during moderate to strong nausea, while a negative correlation was observed during the resting baseline. The results obtained by Kim and Sclocco showed a reduction in HF power and an increase in the HR, associated with alterations in brain activity in specific regions. Molefi et al. confirmed these findings, also reporting an increase in regularity (i.e., lower entropy) and a reduction in chaos in the HRV signal during nausea.

Similarly, the connection between posture and the cardiovascular response to MS was the aim of Yokota et al. (2005) [77]. They analyzed 15 healthy subjects (age ranging from 12 to 36 years) by using 60° (peak-peak) left and right tilts at 0.1, 0.2, and 0.4 Hz. The subjects involved observed the virtual scene while standing on a stable platform with their eyes covered. They found no effect of the visual stimulus on postural sway or HRV in low susceptibility subjects but significant effects in the high susceptibility ones. Moreover, lower frequencies of stimulation led to an increase in LF power and a decrease in HF power of HRV.

The studies by Himi et al. (2004) [78] and Emoto et al. (2008) [79] induced MS through the projection of videos showing oscillating and vibrating images, respectively, on television displays. Both identified the LF/HF ratio as a meaningful parameter for assessing the relationship between HRV and VIMS, highlighting how intense visual stimulation disrupts the classically interpreted sympatho-vagal balance in favor of sympathetic activity. Himi et al. compared autonomic nervous system responses during the viewing of an oscillating video in 17 healthy young volunteers. Eleven participants developed nausea, showing an increased HR and a trend toward a higher LF/HF ratio, while those without nausea exhibited higher and stable LF/HF values from the beginning. On the other hand, Emoto et al. investigated the effect of the visual field angle on VIMS by exposing 15 healthy subjects to stable and vibrating videos on a high-resolution display. Based on SSQ scores and physiological measurements (ECG and skin temperature), they found that larger fields of view intensified MS, with higher SSQ scores and a significant increase in the LF/HF ratio, particularly pronounced during vibrating video exposure.

The experiments conducted by Holmes and Griffin [80] and Gianaros et al. [81] used an optokinetic drum to generate an illusion of movement and induce nausea. Holmes and Griffin investigated the effect of nausea development on HRV in 40 healthy subjects exposed to optokinetic stimulation (mean age = 22.2 years). The protocol included two phases: an initial 12 min baseline recording of physiological measures, followed by an exposure period of up to 32 min. The mean HR was found to increase with higher sickness ratings and nausea. Gianaros et al. analyzed the relationship between cardiac parasympathetic activity and MS severity, assessing RSA, a parasympathetic index derived from ECG and respiratory signals measured minute by minute. Fifty-nine subjects (25 men, aged 18–34 years) were exposed to a rotating optokinetic drum after a 6 min baseline. MS symptoms, monitored using the Pensacola Diagnostic Index (PDI), were inversely correlated with RSA: greater symptom severity was associated with a progressive decrease in RSA.

The studies by Chu et al. [82] (2013) and Cevette et al. [83] (2014) focused on simulator sickness (SS), a form of MS that occurs during or after simulator use. Chu et al. evaluated the effectiveness of transcutaneous electrical nerve stimulation (TENS) in reducing simulator-induced MS under combined visual and mechanical stimuli. Fifteen healthy men (mean age of 28.6 years) participated in four randomized sessions (control, simulator only, TENS only, TENS + simulator). During simulation, HRV reflected sympathetic activation (increased normalized LF and LF/HF ratio, decreased normalized HF), along with higher HR and worsening symptoms. With TENS, autonomic balance was restored, and symptoms were alleviated. Cevette et al. investigated the effect of oculovestibular recoupling (OVR), achieved through galvanic vestibular stimulation (GVS), on autonomic responses during a flight simulation. Twenty-nine healthy participants (18 men, 11 women; mean age 27.1 years) experienced visual vection induced by a prerecorded flight video projected onto a 180° screen, with either synchronized OVR (GVS) or a placebo tactile stimulus. The results showed that OVR stabilized both gastric activity and autonomic balance: in the control condition, HF power decreased significantly, whereas with OVR, HF values remained unchanged.

#### 3.2.2. Mechanical Stimuli to Study MS

Table 2 shows the studies employing mechanical stimuli (such as rotary chair, rotating platform, and treadmill) to study MS. It can be observed that these types of stimuli were employed in eleven studies included in this systematic review. Regarding time-domain HRV features, mHR was employed five times and the RMSSD three times, while the power in both LF and HF bands, as well as the normalized LF and HF power and the LF/HF ratio were employed by almost all the studies as frequency-domain features. In addition, VLF power and PTOT were also adopted in some of the included studies. Finally, it can be observed that the sample size was heterogeneous, ranging from 8 to 109 subjects, while the mean age ranged between 18 and 30 years old.

Among the eleven studies reviewed, three found no correlation between HRV parameters and MS: Mullen et al. (1998) [89], Foster et al. (2020) [91], and Westmoreland et al. (2007) [92].

- Mullen et al. investigated the transfer relationship between instantaneous lung volume and HR in 18 healthy volunteers exposed to a rotating chair with prism glasses and manual/head-movement tasks. No HR increases were linked to MS.
- Westmoreland et al. studied 11 Air Force cadets exposed to Coriolis acceleration in a flight simulator under different head positions and eye conditions. HRV indices (LF, HF, LF/HF) showed no significant changes, though HR and EDA increased, particularly in women.
- Foster et al. examined “sopite syndrome” (MS without nausea, marked by drowsiness and lethargy) in 23 participants exposed to slow sinusoidal platform motions. Despite symptoms and cutaneous vasoconstriction, HRV parameters (RMSSD, LF, HF, LF/HF) did not differ significantly across conditions.

While Westmoreland et al. used Coriolis illusions to evaluate the effect of the head position and eye condition on sympathetic activation, Linjie Wang et al. (2017) [84] employed Coriolis acceleration to train 26 male Chinese astronaut candidates (mean age 22 years). ECG, EGG, and visual sensory data collected before, during, and after training showed significant changes in PTOT, VLF, LF, HF, and LF/HF in the control group, but not in the astronaut trainees. Comparisons across studies highlight that the HRV parameters identified by Wang et al. are particularly sensitive in detecting pre- vs. post-training differences. This aligns with findings from Chu et al. [85]. Zhao et al. [86], Tu et al. [87], and Rui Wang et al. [88], and all emphasize training or behavioral interventions in mitigating MS symptoms. Tu et al. (2021) [87] investigated the effects of yelling intervention on the symptoms and autonomic responses in MS by administering the Coriolis stimulation through a rotary chair (five 1 min stimuli with 1 min rest after each stimulus) to 42 healthy subjects (mean age of 26.6 years). As a result, PTOT, LF, and HF, expressed in the natural logarithmic form, increased during rotational stimuli. After rotation with abdominal straining, no significant effects on HRV parameters were observed in subjects susceptible to MS. In contrast, non-susceptible subjects showed a significant reduction in the normalized LF relative to total power (excluding the VLF component, LF%), along with a trend toward a lower LF/HF ratio and higher HF and HF%. Differently, Coriolis acceleration was used by Wang et al. (2017) to train 26 future Chinese male astronauts (mean age = 22 years) [88]. ECG, electrogastrography, and other eye-related information were acquired before, during, and after the training and showed that the main effect of the test period was found in PTOT, VLF, LF, HF, and LF/HF for control subjects and not in the future astronauts. A rotary chair to induce MS was used by Chu et al. (2012) to study the effect of transcutaneous electrical nerve stimulation on MS in 15 male subjects (mean age of 23.8 years) [85]. Each experimental session was divided into four phases during which physiological parameters were recorded: a control phase, a rotation phase, a phase with electrical stimulation alone, and finally a combined phase with both stimulation and rotation. A 30 min recovery phase followed. HRV analysis showed that rotation suppressed the normalized HF component (HF/PTOT) and increased LF/PTOT, with a progressive rise in the LF/HF ratio. A similar aim was pursued in the study by Zhao et al. (2021) [86], which evaluated the effectiveness of transcutaneous electrical acupoint stimulation (TEA) against MS induced by a rotary chair, in a cohort of 50 healthy subjects (mean age 27.6 years). Each participant underwent two randomized sessions: one with TEA treatment and one with placebo stimulation, separated by at least two weeks. Results showed that, compared to the placebo, HF and LF/HF decreased after MS induction in the acustimulation group, and the stimulation improved participants’ tolerance to MS. Similar findings were reported by Rui Wang et al. (2023) [88], who investigated the effect of hypoxia acclimatization training (HAT) on MS resistance, comparing it with 3D roller training (3DRT). The study involved 48 healthy male military students (mean age 21 years, BMI 22.6 ± 3.3 kg/m^2^), divided into four groups: control, HAT, 3DRT, and combined HAT + 3DRT. MS was induced using a Coriolis chair. HAT consisted of daily 1 h sessions in a hypobaric chamber simulating a 3000 m altitude for 5 days; 3DRT involved progressive multi-axial roller exercises for 3 min daily for 5 days; the combined group performed both protocols over the same period. Questionnaires were collected to assess subjective symptoms, along with physiological parameters such as blood pressure, HR, HRV, and the pupillary light reflex. The study showed that in the control group, there was an increase in nLF and the LF/HF ratio and a decrease in nHF, in parallel with greater MS severity. By contrast, in the training groups, the opposite pattern was observed: nLF and LF/HF decreased, while nHF increased. Shi et al. (2025) [90] compared two vestibular training methods, an electric rotating chair and a visual-stimulation cage, in 109 students susceptible to MS. Before and after training, MS symptoms were assessed with the Graybiel Score, and physiological parameters were measured, including blood pressure and HRV indices (RMSSD, pNN50, LF, HF, and LF/HF). Results showed that in the electric rotating chair group, HF and RMSSD also decreased significantly, indicating reduced sympathetic nervous system activity. In the visual cage group, Graybiel scores and blood pressure similarly decreased, along with significant reductions in RMSSD, pNN50, and HF, while the LF/HF ratio increased. Among participants with high susceptibility to MS, the visual cage group showed a more pronounced reduction in LF, RMSSD, and pNN50, with statistically significant differences compared to the electric chair group. The last two papers employing mechanical stimuli were preclinical studies, Aitake et al. (2011) [63] and Carnevali et al. (2016) [64]. The first study aimed at determining if there was an association between trait anxiety and nausea in 30 rats, while the latter aimed at investigating relationships between the hippocampal theta rhythm and autonomic nervous activity assessed based on HRV in rats. In Carnevali et al. (2016) [64], an increase in the LF/HF ratio was observed during stimulation, whereas in the second, rotation led to an increase in RMSSD and HF components, indicating strong vagal activation, along with a decrease in the LF/HF ratio.

#### 3.2.3. VR to Study MS

Table 3 shows the studies employing VR to study MS.

Thirteen studies were conducted to investigate MS during a VR experiment. All of the studies included a focus on frequency-domain features, except for three studies, of which one did not perform frequency-domain analysis [99], one analyzed the spectral power of the Blood Volume Pulse signal (BVP PSD) instead of HRV [103], and one study [102] did not report the results of the frequency-domain HRV analysis but instead used composite indices such as the Sympathetic Nervous System Index (SNS) and the Parasympathetic Nervous System Index (PNS), which are derived by combining HRV measures from both the time and frequency domains. The latter also analyzed the Stress Index (SI), calculated from the statistical distribution of RR intervals. Both power and normalized power in the HF and LF bands, as well as the LF/HF parameter, were employed. In addition, the PTOT was also adopted in one work. As for the time-domain analysis of HRV parameters, three studies did not include them [93,95,97], while in others, in addition to the indices used to assess VIMS, the mean of normal-to-normal intervals (MeanNN) and the MAD were also measured. Only six studies also included an investigation of the mHR. Moreover, as can be observed in Table 3, the sample size ranged from 5 to 109 subjects, while the mean age varied from 21 to 30 years old.

Most of the studies reported an increase in LF/HF in subjects who experienced MS. However, the study by Zużewicz et al. (2011) [96] reported a reduction in the value. In detail, in the context of simulated driving tasks, the authors investigated the effect of 1 h-long forklift truck virtual driving on the mechanism of autonomic HR in 24 subjects (mean age = 22.9 years); the MS induction made the mRR increase and the LF/HF decrease in the group prone to MS.

An increase in the LF/HF ratio was reported by Watanabe et al. [94] in 2007 and by Lin et al. in 2011 [95] and by Chun-Ling Lin in 2013 [97]. Watanabe’s study, conducted on 22 healthy participants (9 males, mean age 24.39 years), investigated whether a predictive visual signal of yaw and pitch accelerations could influence autonomic nervous system activity during passive translation in a VR environment. Results showed a significant increase in the LF/HF ratio in the absence of predictive cues, while presenting the signal 3500 ms before the event stabilized the ratio.

In 2011, Chin-Teng Lin [95] and Chun-Ling Lin in 2013 [97] explored the relationship between HRV and MS severity, focusing on how behavioral self-regulation might modulate this interaction in a passive driving task. Both studies used a 360° VR driving simulator combined with a six-degrees-of-freedom motion platform equipped with a real car in an immersive lab. The 2011 experiment [95] involved 5 healthy volunteers, while the 2013 study [97] included 26 participants (15 men, 11 women), with 6 later excluded. Data collection in 2011 included ECG, respiration, real-time MS ratings via joystick, and video recordings for counting deep breaths. In 2013, a post-experiment questionnaire was added, and video analysis was extended to detect swallowing and retching. Findings from 2011 showed an increased LF and LF/HF ratio and reduced HF with worsening MS in most subjects, except one whose deep breathing led to opposite patterns. The larger 2013 study, with a more refined analysis of self-regulation, confirmed and expanded these results: 13 subjects showed positive correlations between MS and nLF and LF/HF and negative correlations with nHF, while 7 displayed inverse trends. Malińska et al. (2015) [98] and Ohyama et al. (2007) [93] focused on LF and HF, which, in the second study, are calculated in a normalized form with respect to the total spectral power (LF/PTOT and HF/PTOT). Malińska et al. (2015) [98] evaluated the impact of an hour-long immersion in VR on the mechanisms of autonomic HR control in 19 subjects (mean age = 21.6 years) not susceptible to MS, after a selection through the Coriolis test; the HR and LF are higher during immersion in VR than while watching a stereoscopic 3D movie. Similarity, Ohyama et al. (2007) induced a visual–vestibular conflict through a VR system and assessed subjective symptoms and HRV using a power spectrum analysis on a cohort of 10 young healthy subjects (mean age of 29.7 years) [93]; they found that LF and LF/PTOT increased together with the stimuli, in contrast to HF and HF/PTOT.

In the 2022 study by Park et al. [100], the natural logarithms of VLF and HF components (lnVLF, lnHF) and their ratio (lnVLF/lnHF) were calculated, in addition to HRV time-domain parameters. The aim was to develop a simple and reliable method to assess VR-induced MS and to automatically classify its severity. Twenty-eight subjects (14 males, 14 females, aged 21–34) viewed VR content through both a head-mounted display (HMD) and a 2D screen. Results showed that severe MS symptoms were associated with decreased pNN50, SDNN, and lnHF and with an increased lnVLF and lnVLF/lnHF ratio. In 2023, Hsin et al. [101] analyzed VLF and PTOT components to test three hypotheses regarding SS during a 360° VR learning program. This post-hoc study on 21 medical students also included subjective measures: SSQ, NASA-TLX (Task Load Index) for mental workload, and a Mini-CEX (Clinical Evaluation Exercise) to assess history-taking skills on real patients. Findings indicated that SS was associated with increased VLF (reflecting sympathetic activation) and PTOT (autonomic activation), a higher mental and physical workload, and poorer clinical performance. Moreover, VLF mediated the relationship between SS and clinical performance, while frustration (NASA-TLX) moderated this effect: the more frustrated a student was, the stronger the negative impact of VLF on performance.

In contrast, the 2017 study by Mazloumi Gavgani et al. [99] did not analyze HRV frequency-domain parameters, focusing only on time-domain indices. It aimed to investigate the influence of the optic flow direction on subjective CS symptoms, assessed through a real-time nausea scale and the Motion Sickness Assessment Questionnaire (MSAQ), and on physiological changes. Twelve healthy participants (six males, six females, mean age 27) experienced a 15 min VR roller coaster ride via HMD, once forward and once backwards. Results showed that optic flow direction significantly affected symptoms and physiological markers: during forward rides, RMSSD decreased with increasing nausea, while in backward rides, RMSSD remained stable; SDRR showed no significant correlations in either condition.

Yang et al. [102], Sameri et al. [103], and and Boulic [104] focused on predicting the onset of CS. Yang’s 2023 study explored spatiotemporal brain dynamics and HRV in CS, using these features to predict episodes through a spiking neural network (SNN). Sixty-four healthy participants (29 males, 35 females, aged 18–33) viewed a CS-inducing video. Results showed that SNS and SI indices correlated positively with CS severity. Sameri et al. [103] aimed to develop an implicit, real-time CS-detection system using combined physiological signals (EEG, EDA, HRV) and interpretable AI (XAI), reducing reliance on subjective questionnaires. Twenty-four participants (20 males) were recruited. HRV was extracted from BVP signals, including both time-domain indices and frequency-domain parameters derived from BVP spectral power. Severe CS, indicated by high SSQ scores, was associated with reductions in SDNN, SD1, SD2, S, the SD1/SD2 ratio, and MAD. Tian and Boulic [104] confirmed Yang’s conclusion that ECG alone is unreliable as a physiological marker of CS susceptibility. Their study investigated susceptibility to CS during three-axis rotation and compared objective measures from ECG, EGG, and EEG. Thirty-five participants (17 males, 18 females, mean age 23.3) underwent a “Least Increasing Aversion” (LIA) protocol over four sessions, playing a VR asteroid-shooting game using gaze control. Results showed that although ECG revealed positive correlations among HR, LF, the LF/HF ratio, and CS symptoms, it was not reliable on its own. Instead, EGG proved to be the best physiological marker of nausea, while EEG was useful to assess inter-individual differences in susceptibility.

Finally, Choi et al. [105] studied VR-induced MS in a rehabilitative context where participants walked actively, rather than remaining static. Eleven healthy males (mean age of 23.7) walked on a treadmill at a constant speed of 3.6 km/h for 15 min and were divided into groups based on SSQ scores. Results showed a significant decrease in RMSSD in highly susceptible subjects, especially when using an HMD. The LF/HF ratio increased in susceptible participants with HMD, although its correlation with SSQ was not statistically significant.

#### 3.2.4. Travelling Experience to Study MS

Table 4 shows the studies employing real traveling experience to study MS. Only six studies conducted the experiments during a real traveling experience to induce MS, but they had some important differences. One of them was performed on animals and investigated time-domain features, while the others were focused on time and frequency-domain features. Furthermore, as reported in Table 4, the sample size was small in one paper and larger in the other (18 and 71 subjects, respectively [103]), and the mean age differed (2.7 and 20.1 years, respectively). The most analyzed travel conditions are those related to car trips. Only one study induced MS by exploiting a sea voyage [106]. In one case, the effects of nausea on dogs following a car trip are analyzed [65]. Wang et al. [106] conducted a study on 71 young male subjects (mean age = 20.13 years) through a trip on a boat.

In the first phase of the experiment, participants were selected, and their personal information and basic anthropometric data were collected. In the second phase, they were administered the Graybiel Motion Sickness Questionnaire (GMSQ) and the Nausea Syndrome Rating (NSR), and data on resting energy expenditure and HRV were collected. In the third phase, participants were randomly assigned to two separate ships, and the voyage began. They found that HF decreased with increasing bad feelings, while LF and LF/HF decreased. Studies from Irmak et al. [107] and Henry et al. [108] analyzed the relationship between HRV and MS through experiments on real-car passengers exposed to low-frequency slalom driving (0.2 Hz), though with different aims. Irmak et al. investigated not only the time course of carsickness but also how external versus internal vision (restricted to the cabin) affected symptom development during oscillatory motion. Twenty-four participants sat in the back seat across two sessions, but only 18 (4 women, 14 men, mean age ~26) completed both. Results showed that the LF/HF ratio was significantly correlated with MS severity, but in the opposite direction expected: it decreased as symptoms increased. External vision significantly reduced MS, while internal vision accelerated its onset and intensity. Henry et al. tested real-road driving to examine the effects of lateral acceleration and route predictability on MS and physiological responses. Twenty-four participants (12 men, 12 women, mean age of 39.3) sat in the front seat across two sessions (predictable vs. unpredictable) in a single day. Continuous ECG, EDA, and respiration were recorded, and MS was rated after each slalom using the Car Sickness Rating (CSR). Findings showed that both high lateral acceleration and unpredictability increased CSR, with a significant interaction between the two. Moreover, a higher CSR was associated with a progressive rise in the mean HR, supporting HR as a valid indicator of MS. Schneider et al. [109] analyzed the physiological reactions to unpleasant driving maneuvers on urban, highway, and rural routes, involving 20 participants (13 men, 7 women, 18–31 years old), divided between MS-susceptible and non-susceptible groups. Despite a slight increase in HRV in susceptible subjects, the correlation among SDNN, SMSL, and HR was very low and not significant.

Karjanto et al. [110], on the other hand, evaluated MS during reading in a fully automated vehicle, comparing three conditions: control, peripheral visual feedback (blue LED), and haptic feedback (seat vibrations). The 18 participants (9 men, 9 women, mean age of 28.4) experienced all conditions in 15 min sessions. No significant differences emerged in HRV parameters, but during automated driving, RMSSD values were found to increase. In contrast, more recently, Herbel et al. studied the response of beagle dogs to their first dog trip and found that RMSSD always decreases at the beginning of transports and increases again during the second half of the transport phase, while HR increases at the beginning of the transport [65].

### 3.3. HRV Indices in MS

In this section, we show the statistics regarding the inclusion of time-domain and frequency-domain features across the research included in this systematic review, as well as the protocol for signal acquisition and processing.

#### 3.3.1. Protocol for Cardiac Signal Acquisition and HRV Processing

**Figure 6:**
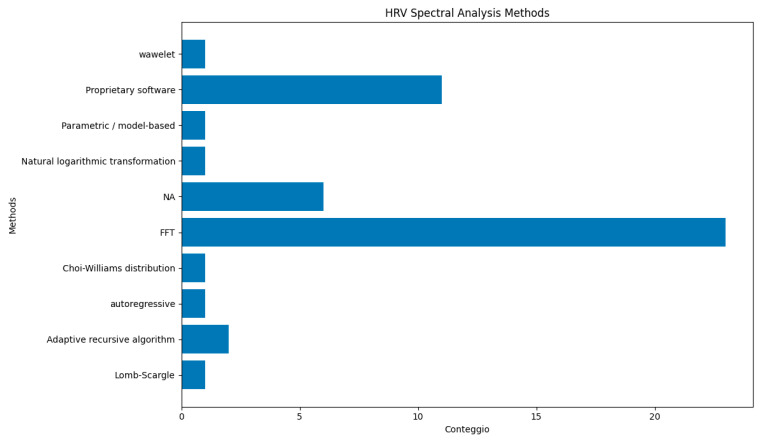
Methods of HRV spectral analysis.

The review of cardiac signal measurement protocols across all articles revealed methodological heterogeneity. This is evident not only in the choice of device used for signal recording but also, and more importantly, in the algorithm employed for signal processing and the method applied for HRV spectral analysis. Regarding the device used, across the included studies, HRV markers were computed from interbeat interval (IBI/RR/NN) series. These intervals were obtained either from ECG recordings (R-peak detection), from PPG/BVP signals (e.g., Empatica E4), or directly from heart rate monitors providing RR intervals (e.g., Polar devices). Furthermore, as graphically illustrated in Figure 6, there is a clear lack of a standardized protocol for calculating the HRV power spectrum. The Fast Fourier Transform (FFT) emerges as the most widely used method, applied in 23 of the studies analysing HRV in the frequency domain, followed by a heterogeneous set of other approaches. Other techniques, such as wavelet transform, Lomb–Scargle, and autoregressive algorithms, are used only once.

**Figure 7:**
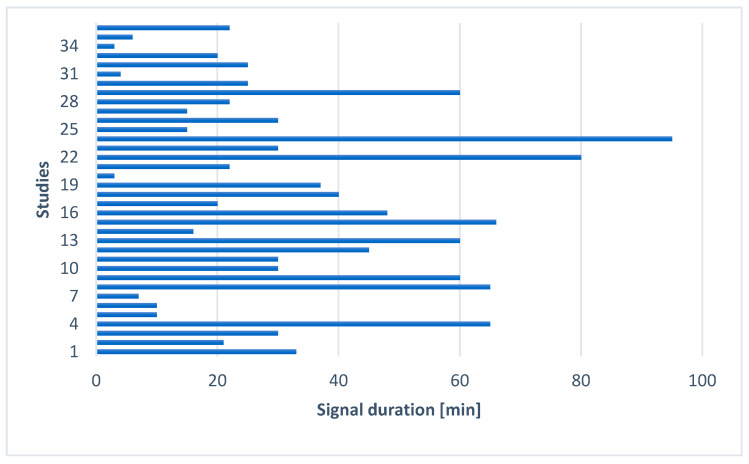
Signal measurement duration.

The lack of homogeneity in measurements is also evident in the signal-recording durations reported across studies, as shown in Figure 7. The lack of homogeneity in measurement procedures is also evident in the recording durations reported across studies. Similar variability is observed in the analysis windows used to compute HRV features. Excluding 9 articles that did not report the analysis window, windows longer than 15 min were used in 4% of cases (2 articles), windows of 5–15 min in 37% (18 articles), and windows shorter than 5 min in 40% (19 articles).

The duration of the analyzed segment significantly influences the physiological interpretation of the derived HRV metrics. Ultra-short recordings (<5 min) may provide acceptable estimates of time-domain markers such as RMSSD, SDNN [111], and SD1 [112]. In the frequency domain, as stated by [33], a recording of approximately 1 min is needed to assess the HF components of HRV, while approximately 2 min are needed to address the LF component. However, although these durations may represent a minimum for estimation, spectral indices derived from <5 min recordings are generally less robust than standard 5 min analyses and remain method- and context-dependent (e.g., stationarity, breathing pattern, and the chosen PSD estimator). In fact, according to [33], short-term (5 min) recordings are appropriate for standardized frequency-domain analysis under controlled conditions, particularly for estimating HF and LF components associated with vagal and baroreflex modulation [113]. Long-term recordings are required to quantify very-low- and ultra-low-frequency HRV components. These bands capture slow fluctuations that are thought to be influenced by longer time-scale regulatory and contextual factors (e.g., thermoregulatory and humoral processes), although their physiological interpretation is less specific and remains partly debated.

Among the studies that use ultra-short-term recordings, seven calculate LF power for epochs lower than 2 min. Such ultra-short segments provide limited spectral resolution, particularly within the LF band (0.04–0.15 Hz), where only a small number of oscillatory cycles can be captured. This limits comparability with studies that employ longer analysis windows. In [88], for example, the authors selected the 90 s segment before, during, and after the rotary chair test. Even if they focused on HRV spectral features for an interval of 90 s, they declare that the time window is relatively short for the spectral analysis.

For real-time applications, time-domain indices based on short recordings, particularly RMSSD and mHR, appear to be the most practical and robust choices. Regarding frequency-domain and more complex nonlinear indices, they generally require more stable and standardized acquisition conditions, conditions that can be difficult to maintain when using ultra-short windows or noisier wearable and contact-free systems.

#### 3.3.2. Included HRV Parameters

*Time-domain parameters*.

**Figure 8:**
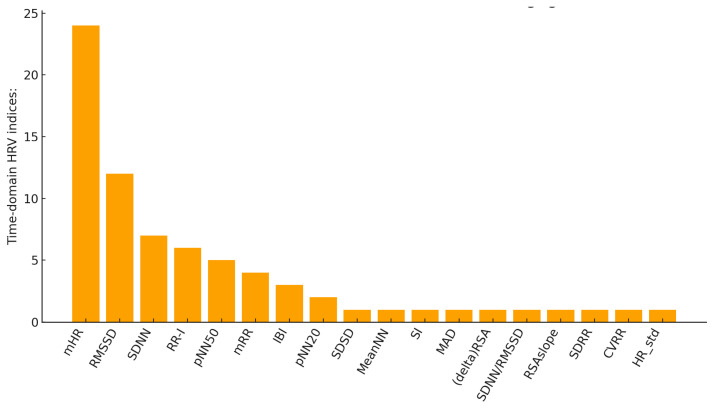
Occurrences of the time-domain features across the included studies.

Figure 8 shows the frequency of use of temporal parameters in the studies included in this review, although time-domain analysis exhibits a low occurrence across all the papers. From the analysis of the indices, it emerges that mHR is the most frequently used in the considered articles, appearing in 24 studies. This is followed by the ‘standard’ time-domain parameters, such as RMSSD and SDNN, which provide direct information on total and parasympathetic variability. However, it is noted that in some studies, parameters that combine the basic time-domain indices are also analyzed, to obtain a view focused on specific aspects of autonomic control, such as MAD, SI, and RSAslope.

*Frequency-domain parameters*.

**Figure 9:**
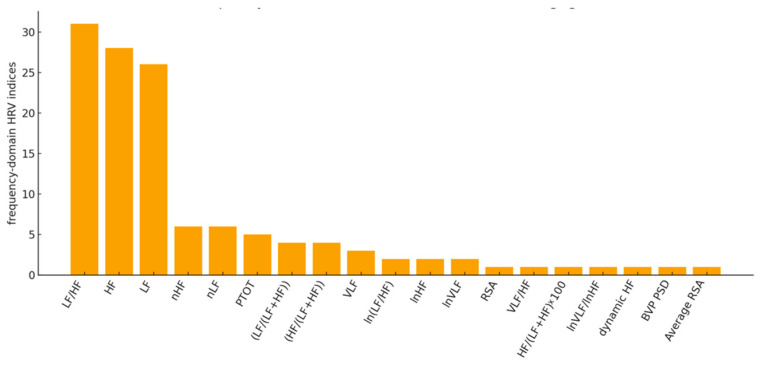
Occurrences of the frequency-domain features across the included studies.

Frequency-domain features were the most frequently employed in this context (Figure 9). In particular, the LF/HF ratio, which represents the Sympatho-Vagal Balance (SVB), has been the most widely used among the HRV indices, showing up in 31 of the 48 articles, followed by the power in HF and LF bands. This appears to be in accordance with the most widespread literature on the HRV indices used in clinical practice [33,114].

*Overall contribution of HRV indices to MS*.

**Figure 10:**
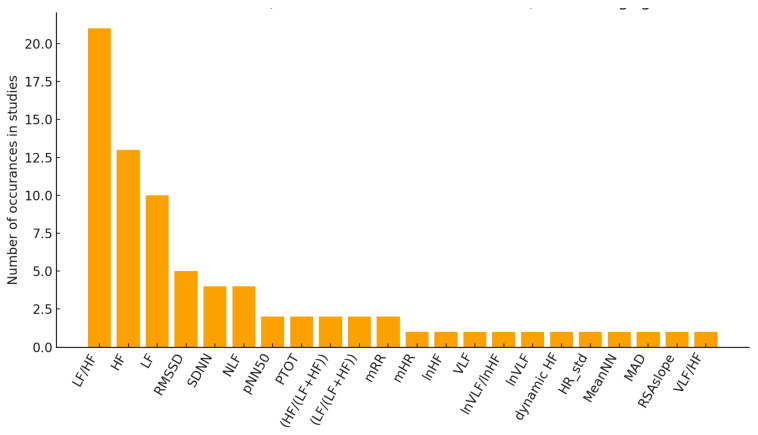
Chart representing the cumulative frequency of HRV indices with a higher correlation with MS.

Finally, the chart in Figure 9 shows how many times each feature is identified as the main one to represent the connection between HRV and MS: LF/HF and HF are the most representative, thereby confirming the previous results shown in Figure 10 and the literature on the most widespread HRV indices [33,114].

#### 3.3.3. HRV Indices in Relation to the Method of MS Induction

Below is an analysis of the variations in HRV indices in relation to the increase in MS symptoms experienced by the study participants.

*Variation of HRV indices due to visual stimuli*.

From the analysis related to the variation in HRV indices due to visual stimuli emerges that the HRV parameters most frequently varying in the presence of MS symptoms are the LF/HF ratio and the HF component. In particular, HF is shown to systematically decrease in all studies where it is analyzed, while the LF/HF ratio increases in subjects susceptible to MS, with the exception of a single study [82]. In one case, the decrease in HF is accompanied by peaks of dynamic HF [73], corresponding to the worsening of nausea. Moreover, in one of these studies, nonlinear HRV indices are also analyzed to assess the complexity and dynamism of the signal.

*Variation in HRV indices due to virtual reality stimuli*.

Also in this case, as MS symptoms increase, the LF/HF ratio is the HRV index that undergoes the greatest variations: specifically, it systematically increases, except in one study [96]. In addition to the standard HRV indices, in one of the studies [103] included in this category, a nonlinear HRV analysis is performed using the Poincaré plot analysis, which highlights a decrease in the S, SD2, and SD1/SD2 indices with increasing symptoms, whereas in another case [102], composite parameters such as SNS and SI are examined, which instead show an increase.

*Variation in HRV indices due to mechanical stimuli*.

From the analysis, and in line with the findings from the previous cases, the HRV features that show the most pronounced variations in relation to the severity of MS symptoms are the LF/HF ratio and the LF and HF components.

*Variation in HRV indices due to real travel experiences*.

In this case, although the number of studies included in this category is smaller than in the others, the LF/HF ratio shows a decrease in [106,107], while the HF component decreases as symptoms increase [106].

*Overall variation in HRV indices in relation to MS symptoms*.

**Figure 11:**
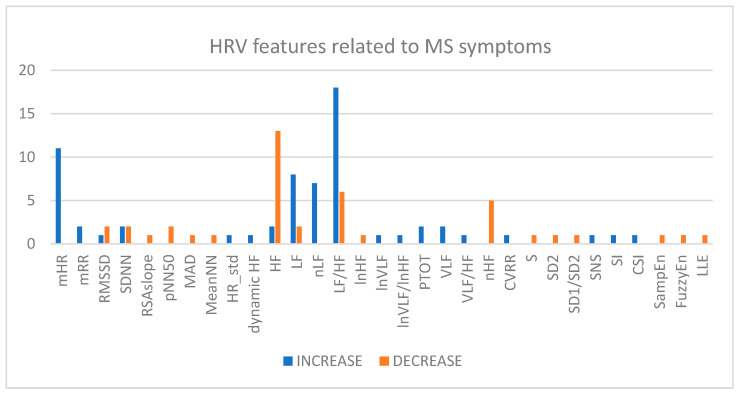
Overall changes in HRV indices.

Finally, Figure 11 presents an overall overview of the variations in HRV characteristics in relation to the intensification of MS symptoms. The graph shows that the features most affected in the presence of MS symptoms are the LF/HF ratio, which in most cases is increased; the HF and LF components (and their normalized versions), which respectively show a decrease and an increase, and the mean HR, which generally tends to increase as symptoms worsen.

In summary, it is worth highlighting that the variation of HRV parameters does not depend on the kind of stimulus but on the MS itself.

## 4. Discussions

To the best of our knowledge, this is the first systematic review to summarize the main findings regarding the connection between HRV indices and MS. There has been a significant increase in attention to MS over the last few years. The articles published on the topic differ in several aspects, such as experimental approaches, the duration of signal registration, and type of analyzed parameters. This diversity highlights the wide range of research being conducted in the field of MS using HRV analysis and the necessity to further investigate the topic under study. Indeed, almost all the authors of the included studies have found an association between their experiments and HRV-related features. The research has included four main ways to induce MS: VR-based, through visual and mechanical stimuli, and with a real trip. The most studied form of MS is VIMS, caused by artificial visual input, followed by CS, linked to immersive VR environments, and SS, observed in simulator users, where MS can result from a combination of mechanical and VR stimuli. With the rise of autonomous vehicles, research is also increasingly focusing on MS induced by their use and on the impact of performing cognitive or visual tasks during travel.

Regarding the study populations, sample sizes ranged from 5 to 119, with mean ages mostly between 24 and 27 years. Thus, the sample size is heterogeneous and limited attention to older adults and those over 40. Frequency-domain features have been the most investigated in this context. In particular, the LF/HF ratio, which represents the SVB and is recognized as a proved tool to assess cardiovascular autonomic regulation [115], and the HF, which reflect the respiratory loop and is a dominant parasympathetic component, are highly sensitive to MS. LF and VLF power, which represent the baroreflex and thermoregulatory loop, have been rarely indicated as the most sensitive. As far as the SVB is useful to quantify the changing relationship: its increase reflects a shift in sympathetic dominance regarding a stressful condition and higher HR; conversely, its decrease corresponds to a parasympathetic dominance with a decrease of cardiac output and HRV during a less stressful situation. The results regarding the relationship between LF/HF and MS are not unanimous in the reported studies [64,96,106,107], but most of them reported an increase during stimulations. The studies focused on healthy individuals, allowing a comparison with standard HR variations. Analyzing healthy subjects helps establish reference values for HRV measurements and better understand changes in those susceptible to MS.

During MS experiments involving various types of stimulation, such as visual, mechanical, or real-world scenarios, subjects undergo physiological changes as they adapt to the stimuli [116]. These changes can affect the SVB and result in alterations in HRV. As far as the HF parameter is concerned, it is influenced by changes in breathing patterns [51] and holds particular interest in the context of MS, as it becomes evident that alterations in breathing, such as an increased breathing rate, occur in response to the feeling of sickness associated with the condition.

Across the included studies, a recurrent cross-study pattern emerges, despite differences in the MS induction paradigms, recording duration, and HRV processing methods. Most studies converge in demonstrating that worsening MS symptoms are associated with a shift toward sympathetic predominance and vagal withdrawal. This pattern is most consistently reflected in an increase in the LF/HF ratio and a decrease in HF power, while the mHR increases. Specifically, this trend was observed in visual, mechanical, VR, and real-world travel paradigms, suggesting that the autonomic response is more closely related to the severity of MS than to the specific stimulus used to induce it. At the same time, the presence of opposing or null findings indicates that protocol heterogeneity, differences in subject susceptibility, and variation in HRV metrics may substantially influence the reported associations.

During visual stimuli, an increase in MS has been consistently associated with elevated measures of mHR, nLF, and LF/HF parameters in multiple studies [66,78,79,80], while HF tends to decrease [72,74,83]. Similarly, mechanical stimulation has shown an increase in LF/HF after inducing MS [63,85,88,90] while an opposite tendency is clearly shown in two other studies [64,86].

These variations have often been observed simultaneously [73,76,77,82]. Some studies also examined mitigation techniques for MS, reporting a reversal of these trends: after applying the correction, LF/HF values decreased while HF increased [70,71,82,83]. VR experiments have also demonstrated similar increases in frequency-domain parameters [93,94,95,97,98,104,105], whereas two of the studies reported an opposite trend [96,101]. Among these, some studies also explore some time-domain measures [96,98,99,100,103,105]. Also, nLF is reported in some articles. Among these, there are also studies in which the authors were unable to identify correlations between HRV and MS [89,91]. Focusing on HF power, all the studies included in our analysis reported a decrease in HF, with the exception of two [64]. Both of these studies used mechanical stimulation to induce motion sickness and found an increase in HF at the end of the rotational stimulus. Specifically, Tu et al. [87] reported a concurrent rise in LF and HF (log-transformed) from pre- to end-of-rotation, suggesting a global increase in spectral power during acute exposure rather than a selective shift toward vagal dominance. Carnevali et al. [64], using a rat model with species-specific HRV frequency bands, observed an increased HF together with a decrease in the LF/HF during prolonged provocative motion, a pattern consistent with a bradycardic/vagal response that may not be directly comparable to human protocols.

Regarding the LF/HF ratio, many studies report an increase during MS exposure, but this directionality is not universal and depends on the study context.

In intervention paradigms, autonomic stimulation can abolish or invert the expected pattern (e.g., TENS eliminated the simulator-sickness-related LF/HF increase, while TEA decreased LF/HF compared with sham in a rotary-chair model) [82,86]. In addition, rodent provocative-motion studies use species-specific HRV bands and may show LF/HF reductions [64]. Two human studies indicate that LF/HF can decrease depending on the exposure duration and on how time vs. sickness severity is modeled: Zużewicz et al. reported a late-exposure reduction in the LF/HF in susceptible participants during prolonged simulator driving [96], and Irmak et al. found that the LF/HF decreased with increasing nausea severity (MISC), with time exerting a smaller, separable effect [107]. Consistent with this pattern, Wang et al. [106] reported that the severe seasickness group showed a significant reduction in the LF/HF during the voyage relative to baseline/recovery, together with reduced sympathetic activity and increased vagal activity.

Slight evidence has also been found regarding the association between MS and time-domain variables such as the average duration of the RR intervals and the RMSSD. A decrease in these variables is indicative of a shift towards sympathetic dominance, whereas increased values suggest a shift towards parasympathetic dominance [87]. Although no significant evidence of a connection between MS and mHR was found in the case of mechanically induced MS [89], a decrease in mRR with MS was found in the case of VR-induced MS [96]. This could be because VR-induced MS may contribute to visual vestibular mismatch events, which can affect the HRV regulation. Given the correlation between mRR and mHR parameters, this suggests the need for further exploration and investigation of the RR and HR trends in different types of MS experiments, in order to assess the difference in heart rhythm control across different stimuli. In the case of travelling experiences, RMSSD has shown an initial decreasing phase followed by an increasing phase [81], while two studies show a decrease in HRV parameters in the frequency [106,107]. Overall, it has been observed that time-domain HRV indices generally decrease under conditions of prolonged stress, whereas frequency-domain indices show significant changes in response to short-term stress.

In three of the studies conducted in recent years and included in this review [76,102,103], complex metrics, such as entropy, chaos, and indices derived from Poincaré analysis, are also examined to clarify the complexity of the regulatory mechanisms of heart rhythm in individuals affected by MS. In particular, study [76] highlights that, in the presence of MS symptoms, entropy values decrease in association with an increase in the LF/HF ratio and a decrease in HF.

This is consistent with the findings of the majority of studies: in the presence of MS, a reduction in the complexity of the HRV signal and vagal tone is observed, accompanied by activation of the sympathetic system. Consequently, future research could consider additional HRV features to further investigate their relationship with MS.

Moreover, the studies have focused mainly on a young and healthy population under the age of 30. It would be of interest to investigate HRV features in studies involving older or pathological subjects, e.g., Parkinson’s disease [77,117].

Furthermore, the application of ML algorithms is anticipated in the study of MS, as already done in some recent research works [118,119,120,121,122,123]. The use of ML and more advanced predictive models could be a valuable tool to support and make further advance in the study of MS, as already done for several other biomedical engineering applications [124,125,126,127,128,129,130].

## 5. Conclusions

This systematic review confirms that the analysis of HRV can support the study and investigation of both the status and functions of cardiac autonomic modulation associated with MS, as HRV indices reflect the sympathetic and parasympathetic responses of individuals to external stimuli and environmental conditions. Across most studies, frequency-domain indices, particularly the LF/HF ratio and HF power, showed the strongest associations with MS symptoms. In general, MS is characterized by an increase in the LF/HF and a decrease in HF, suggesting a shift toward sympathetic dominance and reduced vagal tone. These alterations were observed consistently across visual, mechanical, VR-based, and real-world paradigms, although some studies reported opposite or null results.

Regarding the temporal analysis, the results indicate that the mean HR tends to increase as symptoms worsen, while RMSSD tends to decrease during MS; however, the evidence here is weaker and less consistent compared to frequency-domain features. More advanced metrics (e.g., entropy and Poincaré-derived indices) suggest that MS is associated with a reduction in HRV signal complexity, further supporting the interpretation of impaired parasympathetic modulation.

Future research should address the limitations that characterize the current body of literature. The studies included in this review vary substantially in the design, population size, methodology, and unbalanced gender composition of the analyzed cohorts: most were conducted in young, healthy subjects. This heterogeneity partly explains the different findings, which may limit the generalizability of HRV findings in individuals with MS. Because HRV metrics are known to be influenced by sex differences, future studies should look for more balanced analyses. In addition, a higher degree of standardization in HRV acquisition and analytical procedures is needed. This includes clearer reporting and justification of the recording duration and length of signal analyzed, preprocessing methods, and the selection and physiological interpretation of specific HRV indices. Enhancing these aspects would facilitate comparisons across studies and improve the overall robustness and interpretability of the summary. A key challenge in this field is separating MS-related autonomic responses from non-specific arousal, anxiety, cognitive load, and experimental-task effects, which may also influence HRV. In this context, HRV-informed virtual patient models may enable the in silico simulation of MS responses and systematic testing of conditions potentially associated with MS onset and severity, removing confusing factors.

In summary, HRV, particularly the LF/HF and HF indices, appears to be a sensitive marker of autonomic alterations during MS, but further research is required to establish their reliability and predictive value across different contexts, confounding factors, and populations.

## Figures and Tables

**Table 1:** Studies employing visual stimuli to study MS. ANS: Autonomic Nervous System; CSI: Cardiac Sympathetic Index; CVI: Cardiac Vagal Index; CVRR: Coefficient of Variation of the RR series; ECG: electrocardiography; EGG: electrogastrography; FFT: Fast Fourier Transform; FuzzyEn: Fuzzy Entropy; HF: high frequency; HR: heart rate; HRV: heart rate variability; IBI: interbeat Interval; LLE: Largest Lyapunov Exponent; LF: low frequency; ML: machine learning; MRI: magnetic resonance imaging; MS: motion sickness; MSSQ: Motion Sickness Susceptibility Questionnaire; mHR: mean heart rate; NA: not available; OVR: Oculovestibular Recoupling; PDI: Pensacola Diagnostic Index; pNN20, pNN50: the proportion of NN50 divided by total number of NNs; RMSSD: root mean square of successive differences; RR-I: R-R interval; RSA: respiratory sinus arrhythmia; SampEn: sample entropy; SDNN: standard deviation of NN intervals; SS: Simulator Sickness; SSCQE: Single-Stimulus Continuous Quality Evaluation; SSQ: Simulator Sickness Questionnaire; TENS: Transcutaneous Electrical Nerve Stimulation; VASS-SS: Visual Analogue Scales for Sleepiness Symptoms; VIMS: Visually Induced Motion Sickness; VLF: very low frequency, ↓ decrease, ↑ increase.

Ref.	Aim of the Study	Subjects Involved (Males)	Mean Age	Time-Domain Features	Frequency-Domain Features	Device for the Signal Acquisition	HRV Window [min]	SR (Hz)	Signal-Processing Algorithms Adopted	Main Results Regarding the Relationships Between HRV and MS, HRV Features Related to MS
[66]	Identifying the difference in the level of VIMS using a 2D and a 3D stimulus.	39	NA	NA	HF, LF, LF/HF	NA	1	250	FFT	3D movie induces higher levels of MS than 2D. An increased LF/HF is found in 2D video than in 3D.
[67]	Compare the effects of 3D vs. 2D video on subjective visual fatigue, cognitive performance, and autonomic indices, and test the null hypothesis of no differences between groups.	30 (15)	23.1	RR-I, SDNN, HR	lnVLF, lnHF, VLF/HF ratio	BIOPAC ECG100C + MP100 (BIOPAC Systems, Inc., Goleta, CA, USA)	3	500	(1) R-peak extraction using Pan & Tompkins algorithm. (2) Time features were calculated by measuring RR intervals. (3) HRV spectrum was calculated using FFT (Hanning window technique).	3D viewing was associated with a shift toward sympathetic dominance (HF ↓, (LF/HF ↑), increased overall variability (SDNN ↑), and worse cognitive performance; HRV indices correlated with VIMS severity.
[68]	Developing a method to estimate the continuous change in the degree of VIMS by using physiological indices.	41	27	mHR, CVRR	HF, LF, LF/HF	NA	1	NA	NA	A model consisting of physiological indexes can effectively represent VIMS with higher time and quantitative resolution than a subjective score.
[69]	Evaluate whether machine-learning models using multimodal physiology (ECG, EGG, EDA, respiration, body and facial skin temperature) can detect and predict in real time the severity of VIMS, using the SSQ as the reference outcome.	43 (18)	27.52	mHR, RR-I, RMSSD, pNN50	NA	BIOPAC BioNomadix MP160 (Biopac Systems, Inc.) + AcqKnowledge	1	2000	ECG preprocessed with a 60 Hz notch and 1 Hz zero-phase high-pass; QRS detected via fixed-interval cycle detection with manual review of missed beats; artifacts/ectopic beats corrected to form NN intervals; R–R series segmented into multi-epoch windows to compute time-domain HRV (mean HR, SDNN, RMSSD, pNN50).	Cardiovascular measures for detecting VIMS are inconclusive. HR and HRV are only moderately correlated with VIMS, but were relevant parameters in ML analysis: an increase in HR might be reflective of the psychological stress during VIMS.
[70]	Using simulator sickness questionnaire, ECG, and 3D gaze tracking to investigate VIMS.	40 (32)	22.45	NA	HF, LF, LF/HF	Portable ECG system (WEB-5500; Nihon Koden Co., Tokyo, Japan)	NA	NA	Proprietary software	Horizontal and vertical motion in 3D content were each associated with increased VIMS (ECG/HRV changes—e.g., HR ↑, RMSSD ↓); instructing users to fixate a single stable point reduced symptoms during H/V motion.
[71]	Investigating VIMS in the stereoscopic 3D movie using ECG, eye tracking, and simulator sickness questionnaire.	40 (32)	22.45	NA	HF, LF, LF/HF	WEB-5500; Nihon Koden Co.	NA	NA	Proprietary software	LF/HF increase after exposure to the 3D video.
[72]	Studying the response of the ANS and HRV to MS.	19 (0)	29.1	NA	HF, LF, LF/HF	MRI-compatible Patient Monitor (Model 3150, In Vivo Research, Inc., Orlando, FL, USA)	≈1.20	400	Adaptive recursive algorithm	HF decreases when nausea appears and increases before a strong nausea.
[73]	To address inconsistencies in past studies and apply recent adaptive point-process algorithms for estimating dynamic HRV response to illusory motion-induced nausea, by measuring ECG, SSQ, MSSQ, and respiratory rate.	17 (0)	28.4	HR, mHR	LF, HF, LF/HF ratio, LF/LF + HF, HF/LF + HF, dynamic HF	MRI-compatible Patient Monitor, Model 3150, InVivo Research, Inc., Orlando, FL, USA	1.30 (Dinamic) HRV)/ 4 (Static HRV)	400	ECG with MR gradient/RF artifact removal; R-peaks auto-detected with manual review; NN series from successive R-peaks; HR derived from NN; HRV via FFT-based PSD of HR in 4 min windows; dynamic HRV via point-process adaptive model updated every 10 ms (instantaneous/continuous estimates).	Static and dynamic HRV correlated with motion-sickness severity: HF ↓, LF ↑, LF/HF ↑, HR ↑ (sympathetic activation, vagal withdrawal); transient HF bursts (~10–20 s pre-nausea) suggest anticipatory vagal modulation.
[74]	To reveal the brain circuitry underlying autonomic nervous system responses, specifically cardiovagal modulation, associated with nausea.	21 (0)	28.4	mRR	HF, LF	Model 3150, InVivo Research Inc.	5	400	Adaptive recursive algorithm	HF decreases after the induced MS.
[75]	To evaluate sympathetic and cardiovagal modulation (via HF-HRV and related indices) acquired synchronously with fMRI during nauseogenic visual stimulation inducing vection in motion-sickness-susceptible individuals.	25 (0)	28.4 and 25	mRR	HF	Model 3150, InVivo Research Inc.	4	400	Parametric/model-based	mRR increases in nausea-prone subjects after stimulus, while HF decreases.
[76]	To examine nonlinear HRV metrics (SampEn, FuzzyEn, LLE, CSI, CVI) alongside time- and frequency-domain indices as potential markers of motion-sickness-induced nausea.	14 (2)	26.7	mHR, SDNN, RMSSD, SDNN/RMSSD ratio	lnLF/HF, nLF, nHF	BioSemi ActiveTwo system (Biosemi B. V., Amsterdam, The Netherlands)	5	256	(1) 5 min epoch extraction. (2)R-peak detection using the Pan-Tompkins algorithm. (3) HRV spectrum via Lomb-Scargle periodogram.	HRV features correlated with motion-sickness severity: HR, SDNN, LF, LF/HF, and CSI increased, while HF, SampEn, FuzzyEn, and LLE decreased with nausea intensity—indicating sympathetic dominance and reduced system complexity.
[77]	HRV features correlated with motion-sickness severity: HR, SDNN, LF, LF/HF, and CSI increased, while HF, SampEn, FuzzyEn, and LLE decreased with nausea intensity—indicating sympathetic dominance and reduced system complexity.	15 (12)	12 to 36	NA	nHF, nLF, LF/HF	Multitelemeter system (NihonKoden WEB-5000) and Maclab systems	3	1000	FFT	nLF and LF/HF increase with the stimulus, while nHF decreases.
[78]	To compare autonomic responses between subjects experiencing nausea and those without, by measuring EGG, ECG, palmar and forehead perspiration, digital blood flow, and thoracic respiratory movements.	17 (7)	21.4	mHR, RR-I	LF, HF, LF/HF ratio	AB651J, Nippon Kohden, Japan	1	1000	HR derived from mean R–R intervals averaged over 1 min windows at mid-phase; HRV spectrum computed via FFT using Chart v4 Extension software.	HR and LF/HF ratio increased with nausea intensity, indicating sympathetic activation; subjects resistant to nausea showed higher baseline LF/HF, possibly reflecting a protective autonomic profile.
[79]	To evaluate the effect of viewing angle in vibrating motion pictures on VIMS onset using subjective (SSQ, SSCQE) and physiological (HRV, hand skin temperature) indices, testing the hypothesis that wider fields of view increase motion sickness severity.	15 (1)	32.4	RR-I	LF, HF, LF/HF ratio	Fukuda Densi FM-500 (10-bit digital Holter electrocardiograph recorder)	10	125	R-peaks detected and interpolated R–R intervals computed; HRV power spectra estimated using wavelet-based frequency analysis.	Only the LF/HF ratio significantly correlated with VIMS, increasing during video viewing—suggesting a shift toward sympathetic dominance.
[80]	Assessing the correlation among MS, HR, and HRV	40 (20)	22.2	mHR	HF, LF, LF/HF	NA	NA	NA	Natural logarithmic transformation	An increased level of MS makes the mHR increase. Females suffer from MS more than males.
[81]	To test the hypothesis that a time-related decrease in cardiac parasympathetic activity is associated with nausea and other motion-sickness symptoms during illusory self-motion, by analyzing ECG, respiratory features, and PDI scores.	59 (25)	18 to 34	IBI, RSAslope, (delta)RSA	RSA, Average RSA during rotation	UFI Simple Scope, Model SC2000; UFI	1	1000	IBIs derived from ECG R-spikes and artifact-corrected via Berntson et al. algorithm; RSA computed for each 60 s baseline window using an autoregressive method (Colombo et al.) to estimate spectral power in the respiratory band.	Only RSAslope significantly correlated with motion sickness; a greater minute-by-minute decline in RSA (parasympathetic activity) was associated with stronger nausea, consistent with prior findings in chemotherapy-induced nausea.
[82]	To investigate the effects of TENS on SS, by studying objective parameters (HR, HRV, and salivary stress biomarkers) and subjective parameters (SSQ, VASS-SS, and d2 test of attention).	15 (15)	28.6	mHR	LF, HF, LF/HF ratio, HF/LF + HF, LF/LF + HF	Polar system (RS 800 wrist unit WearLink transmitter, Polar, USA)	NA	NA	(1) HRV was calculated by providing the HR data to the Nevrokard LT-HRV software. (2) Power spectrum analysis was performed using FFT.	During stimulation, HRV indices showed sympathetic activation (LF/LF + HF ↑, LF/HF ↑, HF/LF + HF ↓) and HR ↑ with worsening simulator sickness; after TENS treatment, autonomic balance was restored (HF ↑, LF and LF/HF ↓) with symptom alleviation.
[83]	To quantify the sympathetic and parasympathetic activity through EGG and cardiac inter-beat intervals during flight simulation.	29 (18)	27.1	IBI	LF, HF, HF/(LF + HF) × 100)	BMEYE Nexfin, Amsterdam, The Netherlands	1.04	NA	IBIs continuously derived from peak-to-peak blood pressure; HRV spectra computed via FFT on consecutive 64 s segments with 32 s overlap.	HRV correlated with simulator sickness: without OVR, HF significantly decreased (reduced parasympathetic activity), whereas with OVR, HF remained stable—indicating autonomic stabilization and reduced SS symptoms.

**Table 2:** Studies employing mechanical stimuli to study MS. ECG: electrocardiography; EDA: electrodermal activity; ESS: Epworth Sleepiness Scale; FFT: Fast Fourier Transform; HAT: Hypoxia Acclimatization Training; HF: high frequency; HR: heart rate; HRV: heart rate variability; KSS: Karolinska Sleepiness Scale; LF: low frequency; MS: motion sickness; MSAQ: Motion Sickness Assessment Questionnaire; NA: not available; nHF: normalized high frequency; nLF: normalized low frequency; pNN50: percentage of successive NN intervals differing by more than 50 ms; PTOT: power total; RMSSD: root mean square of successive differences; SSNA: skin sympathetic nerve activity; VLF: very low frequency, ↓ decrease, ↑ increase.

Ref	Aim of the Study	Subjects Involved (Males)	Mean Age	Time-Domain Features	Frequency-Domain Features	Device for the Signal Acquisition	HRV Window [min]	SR (Hz)	Signal-Processing Algorithms Adopted	Main Results Regarding the Relationships Between HRV and MS, HRV Features Related to the MS
[63]	Investigating relationships between hippocampal theta rhythm and autonomic nervous activity assessed by HRV in rats.	8 (8)	NA	mHR	nHF, nLF, LF/HF	Multichannel Acquisition Processor (MAP, Plexon, Dallas, TX, USA)	NA	NA	FFT	A connection between HRV and MS has been found. LF/HF increases after the induced MS.
[64]	Determining if there is an association between trait anxiety and nausea (as reflected by hypothermia) in rats	30 (30)	NA	RMSSD	HF, LF, LF/HF	ART-Gold 4.2	2	1000	FFT	A connection between HRV and MS has been found. During the rotation, RMSSD and HF increase while LF/HF decreases.
[84]	Exploring the different vestibular physiologic response retention patterns after Coriolis acceleration training in student pilots.	26 (26)	21.2 and 22.3	NA	PTOT, VLF, LF, HF, LF/HF	NA	NA	256	FFT	A connection between HRV and MS has been found. The main effect of the test period was found in PTOT, VLF, LF, HF, and LF/HF for control subjects but not in the student pilots.
[85]	Analyzing the effects of transcutaneous electrical nerve stimulation on MS.	15 (15)	23.8	NA	HF, LF, LF/HF, nHF, nLF,	Polar system (RS 800 wrist unit and WearLink transmitter, Polar, USA)	NA	NA	FFT	A connection between HRV and MS has been found. (HF/(LF + HF)) ↓, with the stimulus, (LF/(LF + HF)) and LF/HF ↑ with the stimulus.
[86]	Investigating the effects of transcutaneous electrical acustimulation on MS in healthy subjects.	50 (34)	27.6	NA	nHF, nLF, LF/HF	ECG-01A, Ningbo Maida Medical Device Inc	30	NA	Proprietary software	A connection between HRV and MS has been found. HF/(LF + HF) and LF/HF decrease after the induced MS in the acustimulation group.
[87]	Investigating the effects of yelling intervention on symptoms and autonomic responses in MS.	42 (38)	26.6	NA	PTOT, VLF, LF, HF, LF/HF. Expressed in natural logarithmic form	Miniature physiological signal recorder (TD1, Taiwan Telemedicine Device Company, Taiwan)	9.60	250	FFT	A connection between HRV and MS has been found. PTOT, LF, and HF increase during rotational stimuli.
[88]	To investigate the effects of HAT on the resistance to MS, by assessing ECG, blood pressure and pupillary light reflex.	48 (48)	21	mHR	nLF, nHF, LF/HF, TP	KF2 ECG sensor (Beijing Herserige Technology Co., Ltd., Beijing, China)	1.30	200	HRV was analyzed using the frequency domain algorithm and the tachogram using the FFT parameter model method	As motion-sickness symptoms intensified, nLF and LF/HF ratio ↑, while nHF ↓, indicating sympathetic activation; after training, this pattern reversed (nLF and LF/HF ↓, nHF ↑), suggesting reduced MS susceptibility.
[89]	Measuring the transfer relation between instantaneous lung volume and HR, both before and during moderate MS.	18 (11)	22.3	mHR	NA	Hewlett-Packard, model 7803A	6	360	NA	The authors did not find a connection between mHR and MS.
[90]	Compare electric-rotating vs. visual-motion cage chairs for improving subjective MS symptoms and sympathetic vascular regulation, and identify optimal protocols across MS susceptibility using HR, HRV, and Graybiel scores.	109	≥18	mHR, RMSSD, pNN50	HF, LF, LF/HF ratio	AECG-600D, Nanjing FSYK Software Technology Co., Ltd.	NA	NA	NA	Severe MS was associated with decreased HF, RMSSD, and pNN50 (reduced parasympathetic activity) and increased LF/HF ratio (enhanced sympathetic activity).
[91]	To assess whether imperceptible sinusoidal motion, sufficient to induce Sopite Syndrome without nausea, modulates SSNA and skin blood flow, through analysis of HRV, SSNA, skin blood flow, mean blood pressure, and psychometric questionnaires (MSAQ, KSS, ESS, MSSQ-short).	16 (7)	18 to 30	RMSSD, mHR	LF, HF, LF/HF ratio	PowerLab 16/35, ADInstruments, Sydney, Australia	8–13 min depending on the stimulus	2000	R-waves were detected to generate SSNA auto- and cross-correlation spike histograms (LabChart) for cardiac sympathetic modulation; HRV was analyzed using the LabChart HRV Module with FFT.	This study showed that there was no significant correlation between HRV and sopite syndrome. The authors observed significant variations in SSNA, but no significant changes were found in HRV feature.
[92]	To investigate the influences of head position and eye state on sympathetic activation, by studying HR, HRV, EDA, and respiratory rate.	11 (6)	21	mHR	LF, HF, LF/HF ratio	Biopac MP150, Biopac Systems, Inc., Goleta, CA, USA	~1.41	1000	(1) Interval between successive R-R peaks was used to calculate HR. (2) FFT of R-R interval waveform was used to create a power spectrum and measure HRV	HRV was not a relevant feature for quantifying MS or spatial disorientation following head movement. Meanwhile, HR was good indicator of sympathetic activation during the disorientation, showing a significant increase during that phase.

**Table 3:** Studies employing VR to study MS. ANS: Autonomic Nervous System; BVP: Blood Volume Pulse; CS: cybersickness; ECG: electrocardiography; EDA: electrodermal activity; EEG: electroencephalography; FFT: Fast Fourier Transform; HF: high frequency; HMD: head-mounted display; HR: heart rate; HRV: heart rate variability; IBI: interbeat interval; LF: low frequency; MAD: median absolute deviation; Mini-CEX: Mini Clinical Evaluation Exercise; ML: machine learning; mRR: mean RR-interval; MSSQ: Motion Sickness Susceptibility Questionnaire; NASA TLX: NASA Task Load Index; NA: not available; nHF: normalized high frequency; nLF: normalized low frequency; PNS: Parasympathetic Nervous System; pNN50: percentage of successive NN intervals differing by more than 50 ms; PTOT: power total; RMSSD: root mean square of successive differences; SDRR: standard deviation of RR intervals; SDNN: standard deviation of NN intervals; SDSD: standard deviation of successive differences; SI: Stress Index; SNS: Sympathetic Nervous System; SSQ: Simulator Sickness Questionnaire; VLF: very low frequency; VR: virtual reality, ↓ decrease, ↑ increase.

Ref.	Aim of the Study	Subjects Involved (Males)	Mean Age	Time-Domain Features	Frequency-Domain Features	Device for the Signal Acquisition	HRV Window [min]	SR (Hz)	Signal-Processing Algorithms Adopted	Main Results Regarding the Relationships Between HRV and MS, HRV Features Related to MS
[93]	Assessing subjective symptoms and HRV during VR-induced MS.	10 (9)	29.7	NA	HF, LF, LF/HF, PTOT	ECG telemetry system MT11	7	1000	FFT	The authors find a connection between HRV and MS. LF and LF/PTOT increase together with the stimuli.
[94]	To study the effects of the presentation of a visual sign that warned subjects of acceleration around the yaw and pitch axes in VR on their HRV. The study did not focus on directly inducing or measuring MS, but the results showed a correlation between the sympathetic activation and the symptoms associated to MS.	22 (9)	24.39	RR-I	LF, HF, LF/HF ratio	MP150, BIOPAC system Inc., USA	6	1000	(1) R-peak detection. (2) Calculation of RR intervals to evaluate HRV. (3) HRV spectral parameters were calculated using FFT algorithm	In the absence of predictive cues for upcoming motion changes, the LF/HF ratio increased—indicating sympathetic dominance—associated with motion-sickness symptoms, suggesting HRV as a potential early marker of MS susceptibility.
[95]	Investigating the interplay among HRV, respiration, and the severity of MS in a realistic passive driving task (VR-based).	5	NA	NA	nHF, nLF, LF/HF	NA	5	500	Spike-detection algorithm to detect R peaks; FFT	The authors find a connection between HRV and MS. An increased level of MS makes the nLF and LF/HF increase.
[96]	Determining the effect of a 1 h-long forklift truck virtual simulator driving on the mechanism of autonomic HR regulation in operators.	24	22.9	mRR	HF, LF, LF/HF	Holter Monitor, Medilog Optima apparatus (Oxford Medical Systems, UK)	5	NA	FFT	The authors find a connection between HRV and MS. The MS induction made the mRR increase and the LF/HF decrease.
[97]	Investigating the relationship between HRV and the level of MS induced by simulated tunnel driving.	26 (15)	25	NA	nHF, nLF, log (LF/HF)	NA	5	500	FFT	The authors find a connection between HRV and MS. MS levels increase along with the increase in nLF and LF/HF, whereas nHF decreases along with the increase in the severity of MS.
[98]	Assessing the effect of an hour-long immersion in VR on the mechanisms of autonomic HR regulation among the subjects who were not predisposed to MS.	19 (0)	21.6	mRR	HF, LF, LF/HF	Holter Monitor (Oxford apparatus)	5	NA	Cardioscan system program	The authors find a connection between HRV and MS. The HR and LF are higher during immersion in VR than while watching a stereoscopic 3D movie.
[99]	To assess the influence of visual flow direction on physiological changes and symptoms elicited by cybersickness, through the analysis of HR and cardiac vagal indices, the forehead skin conductance, and responses to the MSSQ.	12 (6)	27	mHR, SDRR, RMSSD	NA	Power Lab 8 data acquisition system + Chart 8.0 software (ADInstruments, Sydney, Australia)	1	1000	HR and the cardiac vagal indices (SDRR and RMSSD) were computed from the ECG trace for each minute of recordings using HRV module of the Chart 8.0 software.	HRV correlated with cybersickness during the forward virtual ride: RMSSD decreased as nausea increased (reduced vagal tone). During the backward ride, RMSSD and SDRR remained stable, with SDRR showing no correlation with nausea in either condition.
[100]	To propose a method for assessing MS induced by watching VR content on a head-mounted display using cardiac features, by comparing HR and HRV derived from ECG signals and SSQ scores during HMD exposure with those obtained during 2D exposure.	28 (14)	26.9	mHR, SDNN, pNN50	lnVLF, lnHF, lnVLF/lnHF ratio	BioNomadix BN-ECG2 + MP160 (Biopac Systems, Inc., Goleta, CA, USA)	5	500	ECG band-pass filtered (5–15 Hz) to minimize artifacts; R-peaks detected via Pan–Tompkins algorithm and converted to NN intervals; time- and frequency-domain HRV metrics extracted in MATLAB 2020b using multi-epoch statistical analysis.	MS severity significantly correlated with an autonomic imbalance: higher symptoms were linked to reduced parasympathetic activity (pNN50 ↓, lnHF ↓), increased sympathetic activity (lnVLF ↑, lnVLF/lnHF ↑), and overall reduced HRV (SDNN ↓).
[101]	To investigate the effects of simulator sickness on autonomic function, mental workload, and learning outcomes by examining HRV, NASA TLX questionnaire, and the Mini-CEX scoring sheet during and after a 360° VR H&P learning program.	28 (20)	24	RR-I	VLF, LF, HF, PTOT, LF/HF ratio	Nexus-4 (MindMedia BV., Herten, The Netherlands)	5	1024	HRV parameters were extracted from a sequence of consecutive 5 min epochs. The power spectrum was quantified using FFT.	HRV correlated with simulator sickness: during the dynamic video phase, VLF and PTOT increased (sympathetic and overall ANS activation), while LF power decreased in subjects experiencing SS, indicating reduced sympatho-vagal balance.
[102]	To explore the spatiotemporal brain dynamics and HRV involved in CS and to use this information to both predict and detect CS episodes, using a deep learning algorithm. The study analyzed HRV, EEG features, MSSQ-short, and SSQ scores.	64 (29)	23	mHR, PNS, SNS, SI, SDNN, RMSSD	NA	Shimmer3 (Shimmer, Dublin, Ireland)	2	512	(1) R-peak detection. (2) HRV parameters calculation by using time-domain analysis.	Some correlations between HRV features and CS prediction are highlighted. SNS and SI are positively correlated with CS symptoms, indicating sympathetic activation, and their measurements contribute to the prediction of CS.
[103]	To detect and measure CS in VR through physiological signals (HRV extracted from blood volume pulse signal-EEG features, breathing rate, EDA, skin temperature), using statistical analysis and ML algorithms, including Poincarè analysis derived metrics.	24 (20)	NA	mHR, IBI, SDNN, RMSSD, SDSD, pNN20, pNN50, MAD	BVP PSD	Empatica E4 wristband (Empatica 2024)	1.5	64	HRV parameters extracted from BVP signals: band-pass filtered (0.5–8 Hz) to remove high-frequency noise; cardiovascular features obtained using the HR Analysis Toolkit.	Significant correlations emerged between HRV and cybersickness severity: lower SDNN, SD2, S, SD1/SD2 ratio, and MAD in the high-CS group indicate sympathetic dominance and reduced autonomic adaptability to VR-induced stress.
[104]	To identify which factor among EEG, EGG, and ECG plays a central role in causing discomfort when experiencing rotations along three axes, by assessing ECG, EEG, and EGG features and SSQ scores.	35 (17)	23.3	mHR, MeanNN, SDNN, RMSSD, PNN50, PNN20	LF, HF, LF/HF ratio	NA	20	NA	Python custom script	HRV features showed partial correlations with cybersickness: HR, LF, and LF/HF ratio positively correlated with CS (sympathetic activation), while MeanNN and HF were negatively correlated (reduced parasympathetic activity). However, HRV alone was insufficient to predict CS.
[105]	To analyze the occurrence of MS when walking on a treadmill in a virtual straight path presented on two types of displays (screen and HMDs) at a constant speed of 3,6 km/h, by studying the ECG and the SSQ.	11 (11)	23.7	mHR, RMSSD	LF/HF	Trigno EKG sensor, Delsys, Natick, MA, USA	1	NA	HR and HRV were extracted using HRV analysis (ANS Lab Tools)	The study confirmed a relationship between HRV and cybersickness: HR and LF/HF ratio increased while RMSSD decreased with worsening CS symptoms, particularly during HMD-assisted walking compared to screen-assisted walking.

**Table 4:** Studies employing a real trip to study MS. CSR: Car Sickness Rating; ECG: electrocardiography; EDA: electrodermal activity; EDR: electrodermal response; FFT: Fast Fourier Transform; GSR: Galvanic Skin Response; HF; high frequency; HR: heart rate; HRV: heart rate variability; LF: low frequency; MISC: Motion Sickness Scale; MS: motion sickness; MSSQ: Motion Sickness Susceptibility Questionnaire; NA: not available; RMSSD: root mean square of successive differences; SDNN: standard deviation of NN intervals; SMSL: Subjective Motion Sickness Level, ↓ decrease, ↑ increase.

Ref	Aim of the Study	Subjects (Males)	Mean Age	Time Feat.000	Freq. Feat.	Device	HRV Window [min]	SR (Hz)	Signal-Processing Algorithms Adopted	Main Results Regarding the Relationships Between HRV and MS, HRV Features Related to MS
[65]	Investigating how Beagle dogs that had not been transported before respond to transport by car.	18 (14)	2.7	mHR, RMSSD	NA	Polar S 810i system (Polar, Kempele, Finland)	5	NA	Kubios HRV Software	The authors find a connection between HRV and MS. RMSSD always decreases at the beginning of transports and increases again during the second half of the transport phase.
[106]	To test the hypothesis that resting energy expenditure, as a major component of thermogenesis, contributes to motion sickness, in association with HRV, and blood ghrelin and leptin levels.	71 (71)	20.1	NA	HF, LF, LF/HF	Biopac MP150 system (Santa Barbara, CA, USA)	5	NA	Frequency domain algorithm developed by Biopac MP150	The authors find a connection between HRV and MS. HF decreases with the increase of the bad feeling, while LF and LF/HF decrease.
[107]	To investigate and model the temporal evolution of MS in a highly dynamic sickening drive, by studying both subjective and physiological responses, through the analysis of MISC, GSR), the head roll, and cardiac parameters.	24 (17)	26.1	mHR	LF/HF ratio	TMSI Mobita amplifier	NA	1000	ECG detrended via 6th-order polynomial fitting; denoised using Sym4 wavelet transform (levels 4–5) with inverse MODWT; R-peaks detected and manually corrected; HR and HRV computed in time domain; spectral analysis performed using the Choi–Williams distribution.	A weak and counterintuitive relationship was observed between HRV and MS LF/HF ratio decreased with increasing MS severity, opposite to the expected sympathetic activation pattern, indicating that LF/HF may not be a reliable marker of MS.
[108]	To evaluate, under real driving conditions, how lateral acceleration and vehicle path predictability influence car sickness incidence and severity, and their relationship with physiological responses via ECG, respiration, skin conductance, and CSR analysis.	24 (12)	39.3	mHR; Standard deviation of HR (Hr_std)	NA	BIOPAC MP160 + Bionomadix (Biopac Systems, Inc.)	0.5	1000	Artifacts detected via Isolation Forest; R-peaks corrected; 50 Hz noise reduced with moving average; baseline drift removed using Butterworth high-pass filter; HRV features extracted in Python (BioSPPy, NeuroKit).	Findings support a relationship between car sickness and cardiac activity: mean HR and HR variability correlated with sickness severity, with stronger symptoms linked to increased cardiac activation, despite inconsistencies in previous literature.
[109]	To identify objective correlations between the autonomic nervous system and the SMSL under real-world traffic conditions, through the analysis of ECG, EDA, EDR, and skin temperature.	20 (13)	23.85	mHR, SDNN	NA	g.tec USBamp, Guger Technologies OG, Austria	5	512	ECG detrended and denoised using 50 Hz IIR notch and 5–50 Hz Butterworth band-pass filters; QRS detected, R-peak artefacts rejected; HRV quantified in 5 min windows.	HR and HRV show no correlation to SMSL in the conditions of this study.
[110]	To investigate the level of experienced MS when reading while being driven in fully automatic driving mode under three different conditions: no intervention, two different types of interventions. The study analyzed ECG and MSSQ scores.	18 (9)	28.4	RMSSD, mHR	HF	NA	5	250	HF component extracted via FFT from the ECG signal.	The study shows no significant correlation between HRV and MS: RMSSD and HF increased when the vehicle was in motion, indicating relaxation rather than a worsening in MS symptoms. The results of the study are inconclusive.

## Data Availability

The original contributions presented in this study are included in the article. Further inquiries can be directed to the corresponding author.

## Abbreviations

ANS	Autonomic Nervous System
BVP	Blood Volume Pulse
CS	Cybersickness
CSR	Car Sickness Rating
CSI	Cardiac Sympathetic Index
CVI	Cardiac Vagal Index
CVRR	Coefficient of Variation of RR intervals
ECG	Electrocardiography
EDA	Electrodermal Activity
EDR	Electrodermal Response
EEG	Electroencephalography
EGG	Electrogastrography
EMG	Electromyography
ESS	Epworth Sleepiness Scale
FFT	Fast Fourier Transform
FMS	Fast Motion Sickness Scale
FuzzyEn	Fuzzy Entropy
GSR	Galvanic Skin Response
GVS	Galvanic Vestibular Stimulation
HF	High Frequency
HMD	Head-Mounted Display
HR	Heart Rate
HR_std	Standard Deviation of HR
HRV	Heart Rate Variability
IBI	Inter-Beat Interval
KSS	Karolinska Sleepiness Scale
LLE	Largest Lyapunov Exponent
LF	Low Frequency
LF/HF	Low Frequency/High Frequency ratio
MAD	Median Absolute Deviation
MeanNN	Mean of NN intervals
Mini-CEX	Mini Clinical Evaluation Exercise
MISC	Motion Sickness Scale
ML	Machine Learning
MRI	Magnetic Resonance Imaging
MS	Motion Sickness
MSAQ	Motion Sickness Assessment Questionnaire
MSSQ	Motion Sickness Susceptibility Questionnaire
mHR	Mean Heart Rate
mRR	Mean RR interval
NA	Not Available
NASA-TLX	NASA Task Load Index
nHF	Normalized High Frequency
nLF	Normalized Low Frequency
OVR	Oculovestibular Recoupling
PDI	Pensacola Diagnostic Index
pNN20	Percentage of adjacent intervals differing by more than 20 ms
pNN50	Percentage of adjacent intervals differing by more than 50 ms
PNS	Parasympathetic Nervous System
PSD	Power Spectral Density
PTOT	Total spectral power (Power Total)
RMSSD	Root Mean Square of Successive Differences
RR-I	R-R Interval
RSA	Respiratory Sinus Arrhythmia
SampEn	Sample Entropy
SD1, SD2	Poincarè plot indices (axes)
SDNN	Standard Deviation of NN intervals
SDRR	Standard Deviation of RR intervals
SDSD	Standard Deviation of Successive Differences
SI	Stress Index
SR	Sampling rate
SMSL	Subjective Motion Sickness Level
SMS	Space Motion Sickness
SNS	Sympathetic Nervous System
SS	Simulator Sickness
SSCQE	Single-Stimulus Continuous Quality Evaluation
SSNA	Skin Sympathetic Nerve Activity
SSQ	Simulator Sickness Questionnaire
SVB	Sympatho-Vagal Balance
TEA	Transcutaneous Electrical Acupoint Stimulation
TENS	Transcutaneous Electrical Nerve Stimulation
TP	Total Power
VASS-SS	Visual Analogue Scales for Sleepiness Symptoms
VIMS	Visually Induced Motion Sickness
VLF	Very Low Frequency
VR	Virtual Reality

## References

[1] Koohestani A., Nahavandi D., Asadi H., Kebria P.M., Khosravi A., Alizadehsani R., Nahavandi S. (2019). A Knowledge Discovery in Motion Sickness: A Comprehensive Literature Review. IEEE Access.

[2] Reason J.T., Brand J.J. (1975). Motion Sickness.

[3] Reason J.T. (1978). Motion Sickness Adaptation: A Neural Mismatch Model. J. R. Soc. Med..

[4] Tal D., Wiener G., Shupak A. (2014). Mal de debarquement, motion sickness and the effect of an artificial horizon. J. Vestib. Res..

[5] Keshavarz B., Golding J.F. (2022). Motion sickness: Current concepts and management. Curr. Opin. Neurol..

[6] Leung A.K., Hon K.L. (2019). Motion sickness: An overview. Drugs Context.

[7] Koch A., Cascorbi I., Westhofen M., Dafotakis M., Klapa S., Kuhtz-Buschbeck J.P. (2018). The Neurophysiology and Treatment of Motion Sickness. Dtsch. Arztebl. Int..

[8] Golding J.F., Gresty M.A. (2015). Pathophysiology and treatment of motion sickness. Curr. Opin. Neurol..

[9] Lackner J.R. (2014). Motion sickness: More than nausea and vomiting. Exp. Brain Res..

[10] Cha Y.H., Golding J.F., Keshavarz B., Furman J., Kim J.S., Lopez-Escamez J.A., Magnusson M., Yates B.J., Lawson B.D., Staab J. (2021). Motion sickness diagnostic criteria: Consensus Document of the Classification Committee of the Bárány Society. J. Vestib. Res..

[11] Bertolini G., Straumann D. (2016). Moving in a Moving World: A Review on Vestibular Motion Sickness. Front. Neurol..

[12] Iskander J., Attia M., Saleh K., Nahavandi D., Abobakr A., Mohamed S., Asadi H., Khosravi A., Lim C.P., Hossny M. (2019). From car sickness to autonomous car sickness: A review. Transp. Res. Part F Traffic Psychol. Behav..

[13] Golding J.F. (2006). Motion sickness susceptibility. Auton. Neurosci..

[14] Kennedy R.S., Drexler J., Kennedy R.C. (2010). Research in visually induced motion sickness. Appl. Ergon..

[15] Khalid A., Prusty P.P., Arshad I., Gustafson H.E., Jalaly I., Nockels K., Bentley B.L., Goel R., Ferre E.R. (2023). Pharmacological and non-pharmacological countermeasures to Space Motion Sickness: A systematic review. Front. Neural Circuits.

[16] Laessoe U., Abrahamsen S., Zepernick S., Raunsbaek A., Stensen C. (2023). Motion sickness and cybersickness—Sensory mismatch. Physiol. Behav..

[17] Caserman P., Garcia-Agundez A., Zerban A.G., Göbel S. (2021). Cybersickness in current-generation virtual reality head-mounted displays: Systematic review and outlook. Virtual Real..

[18] Recenti M., Ricciardi C., Aubonnet R., Picone I., Jacob D., Svansson H.Á., Agnarsdóttir S., Karlsson G.H., Baeringsdóttir V., Petersen H. (2021). Toward Predicting Motion Sickness Using Virtual Reality and a Moving Platform Assessing Brain, Muscles, and Heart Signals. Front. Bioeng. Biotechnol..

[19] Kim J., Oh H., Kim W., Choi S., Son W., Lee S. (2022). A Deep Motion Sickness Predictor Induced by Visual Stimuli in Virtual Reality. IEEE Trans. Neural Netw. Learn. Syst..

[20] Samuel O., Tal D. (2015). Airsickness: Etiology, Treatment, and Clinical Importance—A Review. Mil. Med..

[21] Perrin P., Lion A., Bosser G., Gauchard G., Meistelman C. (2013). Motion Sickness in Rally Car Co-Drivers. Aviat. Space Environ. Med..

[22] Schmäl F. (2013). Neuronal Mechanisms and the Treatment of Motion Sickness. Pharmacology.

[23] Lawther A., Griffin M.J. (1988). A survey of the occurrence of motion sickness amongst passengers at sea. Aviat. Space Environ. Med..

[24] Paillard A.C., Quarck G., Paolino F., Denise P., Paolino M., Golding J.F., Philoxene B.V. (2013). Motion sickness susceptibility in healthy subjects and vestibular patients: Effects of gender, age and trait-anxiety. J. Vestib. Res..

[25] Muth E.R. (2006). Motion and space sickness: Intestinal and autonomic correlates. Auton. Neurosci..

[26] Cowings P.S., Suter S., Toscano W.B., Kamiya J., Naifeh K. (1986). General Autonomic Components of Motion Sickness. Psychophysiology.

[27] Sztajzel J. (2004). Heart rate variability: A noninvasive electrocardiographic method to measure the autonomic nervous system. Swiss Med. Wkly..

[28] Romano M., Cesarelli M., Bifulco P., Sansone M., Bracale M. (2003). Study of fetal autonomous nervous system’s response by means of FHRV frequency analysis. Proceedings of the First International IEEE EMBS Conference on Neural Engineering, 2003, Capri Island, Italy, 20–22 March 2003.

[29] Cysarz D., Lange S., Matthiessen P.F., van Leeuwen P. (2007). Regular heartbeat dynamics are associated with cardiac health. Am. J. Physiol. Regul. Integr. Comp. Physiol..

[30] Porta A., Tobaldini E., Guzzetti S., Furlan R., Montano N., Gnecchi-Ruscone T. (2007). Assessment of cardiac autonomic modulation during graded head-up tilt by symbolic analysis of heart rate variability. Am. J. Physiol. Heart Circ. Physiol..

[31] Cardoso S., Silva M.J., Guimarães H. (2017). Autonomic nervous system in newborns: A review based on heart rate variability. Childs Nerv. Syst..

[32] Castro L., Loureiro M., Henriques T.S., Nunes I. (2021). Systematic Review of Intrapartum Fetal Heart Rate Spectral Analysis and an Application in the Detection of Fetal Acidemia. Front. Pediatr..

[33] Task Force of the European Society of Cardiology the North American Society of Pacing Electrophysiology (1996). Heart Rate Variability: Standards of Measurement, Physiological Interpretation, and Clinical Use. Circulation.

[34] Kim H.G., Cheon E.J., Bai D.S., Lee Y.H., Koo B.H. (2018). Stress and Heart Rate Variability: A Meta-Analysis and Review of the Literature. Psychiatry Investig..

[35] Cascella M., Quattrone D., Sessa F., Esposito G., Marotta A., Micomonaco M.W., Crispo A., Barberio D., Gagliardi G., Vittori A. (2026). Linking Cancer Pain Features and Biosignals for Automatic Pain Assessment. Cancers.

[36] Santoriello V., Collodel S., Guerrini L., Pescaglia F., Jónsson H., Petersen H., Angelone F., Ricciardi C., Amato F., Romano M. (2025). Exploring Heart Rate Variability During a Postural Control Task using Virtual Reality and a Moving Platform. Proceedings of the 2025 IEEE International Conference on Metrology for eXtended Reality, Artificial Intelligence and Neural Engineering (MetroXRAINE), Ancona, Italy, 22–24 October 2025.

[37] Santoriello V., Ponsiglione A.M., Giugliano C., Buonaguro C., Gallo L., Caggianese G., Cascella M., De Pietro G., Chirico A., Giordano A. (2025). Virtual Reality and Biosignals for Labor Pain Relief: A Pilot Study. Proceedings of the 2025 IEEE International Conference on Metrology for eXtended Reality, Artificial Intelligence and Neural Engineering (MetroXRAINE), Ancona, Italy, 22 October 2025.

[38] Tiwari R., Kumar R., Malik S., Raj T., Kumar P. (2021). Analysis of Heart Rate Variability and Implication of Different Factors on Heart Rate Variability. Curr. Cardiol. Rev..

[39] Sammito S., Böckelmann I. (2016). Factors influencing heart rate variability. Int. Cardiovasc. Forum J..

[40] Hering D., Somers V.K., Kara T., Kucharska W., Jurak P., Bieniaszewski L., Narkiewicz K. (2006). Sympathetic neural responses to smoking are age dependent. J. Hypertens..

[41] Kageyama T., Nishikido N., Honda Y., Kurokawa Y., Imai H., Kobayashi T., Kaneko T., Kabuto M. (1997). Effects of obesity, current smoking status, and alcohol consumption on heart rate variability in male white-collar workers. Int. Arch. Occup. Environ. Health.

[42] Lane J.D., Manus D.C. (1989). Persistent cardiovascular effects with repeated caffeine administration. Psychosom. Med..

[43] Karpyak V.M., Romanowicz M., Schmidt J.E., Lewis K.A., Bostwick J.M. (2014). Characteristics of Heart Rate Variability in Alcohol-Dependent Subjects and Nondependent Chronic Alcohol Users. Alcohol. Clin. Exp. Res..

[44] Järvelin-Pasanen S., Sinikallio S., Tarvainen M.P. (2018). Heart rate variability and occupational stress—systematic review. Ind. Health.

[45] Chalmers J.A., Quintana D.S., Abbott M.J., Kemp A.H. (2014). Anxiety Disorders are Associated with Reduced Heart Rate Variability: A Meta-Analysis. Front. Psychiatry.

[46] Koch C., Wilhelm M., Salzmann S., Rief W., Euteneuer F. (2019). A meta-analysis of heart rate variability in major depression. Psychol. Med..

[47] Granath J., Ingvarsson S., von Thiele U., Lundberg U. (2006). Stress Management: A Randomized Study of Cognitive Behavioural Therapy and Yoga. Cogn. Behav. Ther..

[48] Quintana D.S., Guastella A.J., Outhred T., Hickie I.B., Kemp A.H. (2012). Heart rate variability is associated with emotion recognition: Direct evidence for a relationship between the autonomic nervous system and social cognition. Int. J. Psychophysiol..

[49] Laborde S., Mosley E., Thayer J.F. (2017). Heart Rate Variability and Cardiac Vagal Tone in Psychophysiological Research – Recommendations for Experiment Planning, Data Analysis, and Data Reporting. Front. Psychol..

[50] Young H.A., Benton D. (2018). Heart-rate variability: A biomarker to study the influence of nutrition on physiological and psychological health?. Behav. Pharmacol..

[51] Shaffer F., Ginsberg J.P. (2017). An Overview of Heart Rate Variability Metrics and Norms. Front. Public Health.

[52] Karjanto J., Yusof N.M., Wang C., Terken J., Delbressine F., Rauterberg M. (2018). The effect of peripheral visual feedforward system in enhancing situation awareness and mitigating motion sickness in fully automated driving. Transp. Res. Part F Traffic Psychol. Behav..

[53] Takkouche B., Norman G. (2011). PRISMA Statement. Epidemiology.

[54] Selcuk A.A. (2019). A Guide for Systematic Reviews: PRISMA. Turk. Arch. Otorhinolaryngol..

[55] Sassi R., Cerutti S., Lombardi F., Malik M., Huikuri H.V., Peng C.-K., Schmidt G., Yamamoto Y., Gorenek B., Lip G.Y.H. (2015). Advances in heart rate variability signal analysis: Joint position statement by the e-Cardiology ESC Working Group and the European Heart Rhythm Association co-endorsed by the Asia Pacific Heart Rhythm Society. Europace.

[56] Pham T., Lau Z.J., Chen S.H.A., Makowski D. (2021). Heart Rate Variability in Psychology: A Review of HRV Indices and an Analysis Tutorial. Sensors.

[57] Chattopadhyay S. (2023). The Importance of Time-Domain HRV Analysis in Cardiac Health Prediction. Ser. Cardiol. Res..

[58] Bogdan C., Apostol A., Ivan V.M., Sandu O.E., Petre I., Suciu O., Marc L.-E., Maralescu F.-M., Lighezan D.F. (2024). Heart Rate Variability and Global Longitudinal Strain for Prognostic Evaluation and Recovery Assessment in Conservatively Managed Post-Myocardial Infarction Patients. J. Clin. Med..

[59] Azami H., Li P., Arnold S., Escudero J., Humeau-Heurtier A. (2019). Fuzzy Entropy Metrics for the Analysis of Biomedical Signals: Assessment and Comparison. IEEE Access.

[60] Yokobori Y., Nakane H., Uehara C., Nagasawa T., Mitsuyama S., Ohkawa K., Kario K., Ozawa S. (2023). Temporal relationships among changes in the RR-interval and the powers of the low- and high-frequency components of heart rate variability in normal subjects. Physiol. Rep..

[61] Rahman F., Pechnik S., Gross D., Sewell L., Goldstein D.S. (2011). Low frequency power of heart rate variability reflects baroreflex function, not cardiac sympathetic innervation. Clin. Auton. Res..

[62] Billman G.E. (2013). The LF/HF ratio does not accurately measure cardiac sympatho-vagal balance. Front. Physiol..

[63] Aitake M., Hori E., Matsumoto J., Umeno K., Fukuda M., Ono T., Nishijo H. (2011). Sensory mismatch induces autonomic responses associated with hippocampal theta waves in rats. Behav. Brain Res..

[64] Carnevali L., Andrews P.L., Neumann I.D., Nalivaiko E., Sgoifo A. (2016). Autonomic changes induced by provocative motion in rats bred for high (HAB) and low (LAB) anxiety-related behavior: Paradoxical responses in LAB animals. Physiol. Behav..

[65] Herbel J., Aurich J., Gautier C., Melchert M., Aurich C. (2020). Stress Response of Beagle Dogs to Repeated Short-Distance Road Transport. Animals.

[66] Naqvi S.A.A., Badruddin N., Malik A.S., Hazabbah W., Abdullah B. (2013). Does 3D produce more symptoms of visually induced motion sickness?. Proceedings of the 2013 35th Annual International Conference of the IEEE Engineering in Medicine and Biology Society (EMBC), Osaka, Japan, 3–7 July 2013.

[67] Park S., Won M.J., Mun S., Lee E.C., Whang M. (2014). Does visual fatigue from 3D displays affect autonomic regulation and heart rhythm?. Int. J. Psychophysiol..

[68] Tanaka A., Sugita N., Yoshizawa M., Abe M., Yambe T. (2008). Interpolation of the subjective score of visually-induced motion sickness by using physiological parameters. Proceedings of the 2008 30th Annual International Conference of the IEEE Engineering in Medicine and Biology Society, Vancouver, BC, Canada, 20–24 August 2008.

[69] Keshavarz B., Peck K., Rezaei S., Taati B. (2022). Detecting and predicting visually induced motion sickness with physiological measures in combination with machine learning techniques. Int. J. Psychophysiol..

[70] Wibirama S., Hamamoto K. (2014). Investigation of visually induced motion sickness in dynamic 3D contents based on subjective judgment, heart rate variability, and depth gaze behavior. Proceedings of the 2014 36th Annual International Conference of the IEEE Engineering in Medicine and Biology Society, Chicago, IL, USA, 26–30 August 2014.

[71] Wibirama S., Nugroho H.A., Hamamoto K. (2018). Depth gaze and ECG based frequency dynamics during motion sickness in stereoscopic 3D movie. Ent. Comput..

[72] LaCount L.T., Napadow V., Kuo B., Park K., Kim J., Brown E.N., Barbieri R. (2009). Dynamic cardiovagal response to motion sickness: A point-process heart rate variability study. Proceedings of the 2009 36th Annual Computers in Cardiology Conference (CinC), Park City, UT, USA, 13–16 September 2009.

[73] LaCount L.T., Barbieri R., Park K., Kim J., Brown E.N., Kuo B., Napadow V. (2011). Static and Dynamic Autonomic Response with Increasing Nausea Perception. Aviat. Space Environ. Med..

[74] Kim J., Napadow V., Kuo B., Barbieri R. (2011). A combined HRV-fMRI approach to assess cortical control of cardiovagal modulation by motion sickness. Proceedings of the 2011 Annual International Conference of the IEEE Engineering in Medicine and Biology Society, Boston, MA, USA, 30 August–3 September 2011.

[75] Sclocco R., Kim J., Garcia R.G., Sheehan J.D., Beissner F., Bianchi A.M., Cerutti S., Kuo B., Barbieri R., Napadow V. (2016). Brain Circuitry Supporting Multi-Organ Autonomic Outflow in Response to Nausea. Cereb. Cortex.

[76] Molefi E., McLoughlin I., Palaniappan R. (2023). Heart Rate Variability Responses to Visually Induced Motion Sickness. Proceedings of the 2023 45th Annual International Conference of the IEEE Engineering in Medicine & Biology Society (EMBC), Sydney, Australia, 24–27 July 2023.

[77] Yokota Y., Aoki M., Mizuta K., Ito Y., Isu N. (2005). Motion sickness susceptibility associated with visually induced postural instability and cardiac autonomic responses in healthy subjects. Acta Otolaryngol..

[78] Himi N., Koga T., Nakamura E., Kobashi M., Yamane M., Tsujioka K. (2004). Differences in autonomic responses between subjects with and without nausea while watching an irregularly oscillating video. Auton. Neurosci..

[79] Emoto M., Sugawara M., Nojiri Y. (2008). Viewing angle dependency of visually-induced motion sickness in viewing wide-field images by subjective and autonomic nervous indices. Displays.

[80] Holmes S.R., Griffin M.J. (2001). Correlation between heart rate and the severity of motion sickness caused by optokinetic stimulation. J. Psychophysiol..

[81] Gianaros P.J., Quigley K.S., Muth E.R., Levine M.E., Vasko R.C., Stern R.M. (2003). Relationship between temporal changes in cardiac parasympathetic activity and motion sickness severity. Psychophysiology.

[82] Chu H., Li M.H., Huang Y.C., Lee S.Y. (2013). Simultaneous transcutaneous electrical nerve stimulation mitigates simulator sickness symptoms in healthy adults: A crossover study. BMC Complement. Altern. Med..

[83] Cevette M.J., Pradhan G.N., Cocco D., Crowell M.D., Galea A.M., Bartlett J., Stepanek J. (2014). Electrogastrographic and Autonomic Responses During Oculovestibular Recoupling in Flight Simulation. Aviat. Space Environ. Med..

[84] Wang L., Cao Y., Tan C., Zhao Q., He S., Niu D., Tang G., Zou P., Xing L. (2017). Uncoupling VOR and vestibuloautonomic retention to Coriolis acceleration training in student pilots and control subjects. J. Vestib. Res..

[85] Chu H., Li M.H., Juan S.H., Chiou W.Y. (2012). Effects of Transcutaneous Electrical Nerve Stimulation on Motion Sickness Induced by Rotary Chair: A Crossover Study. J. Altern. Complement. Med..

[86] Zhao Q., Ning B.-F., Zhou J.-Y., Wang J., Yao Y.-J., Peng Z.-Y., Yuan Z.-L., Chen J.D., Xie W.-F. (2022). Transcutaneous Electrical Acustimulation Ameliorates Motion Sickness Induced by Rotary Chair in Healthy Subjects: A Prospective Randomized Crossover Study. Neuromodulation.

[87] Tu M.-Y., Chu H., Lai C.-Y., Chiang K.-T., Huang C.-C., Chin H.-C., Wen Y.-H., Chen C.-L. (2021). Effect of Standardized Yelling on Subjective Perception and Autonomic Nervous System Activity in Motion Sickness. Int. J. Environ. Res. Public Health.

[88] Wang R., Yan Y., Tie Y., Zhang Q., Pan Y., Li S., Fan J., Li C., Li X., Wang Y. (2023). Hypoxic acclimatization training improves the resistance to motion sickness. Front. Neurosci..

[89] Mullen T.J., Berger R.D., Oman C.M., Cohen R.J. (1998). Human Heart Rate Variability Relation Is Unchanged During Motion Sickness. J. Vestib. Res..

[90] Shi L., Zhao J., Lu J., Cao C., Zhang Q., Qiu C., Jin Z., Yan S. (2025). Effects of two kinds of vestibular function training on reducing motion sickness in college students. Front. Neurol..

[91] Foster M., Singh N., Kwok K., Macefield V.G. (2020). Vestibular modulation of skin sympathetic nerve activity in sopite syndrome induced by low-frequency sinusoidal motion. J. Neurophysiol..

[92] Westmoreland D., Krell R.W., Self B.P. (2007). Physiological Responses to the Coriolis Illusion: Effects of Head Position and Vision. Aviat. Space Environ. Med..

[93] Ohyama S., Nishiike S., Watanabe H., Matsuoka K., Akizuki H., Takeda N., Harada T. (2007). Autonomic responses during motion sickness induced by virtual reality. Auris Nasus Larynx.

[94] Watanabe H., Teramoto W., Umemura H. (2007). Effect of predictive sign of acceleration on heart rate variability in passive translation situation: Preliminary evidence using visual and vestibular stimuli in VR environment. J. Neuroeng. Rehabil..

[95] Lin C.T., Lin C.L., Chiu T.W., Duann J.R., Jung T.P. (2011). Effect of respiratory modulation on relationship between heart rate variability and motion sickness. Proceedings of the 2011 Annual International Conference of the IEEE Engineering in Medicine and Biology Society, Boston, MA, USA, 30 August–3 September 2011.

[96] Zużewicz K., Saulewicz A., Konarska M., Kaczorowski Z. (2011). Heart Rate Variability and Motion Sickness During Forklift Simulator Driving. Int. J. Occup. Saf. Ergon..

[97] Lin C.L., Jung T.P., Chuang S.W., Duann J.R., Lin C.T., Chiu T.W. (2013). Self-adjustments may account for the contradictory correlations between HRV and motion-sickness severity. Int. J. Psychophysiol..

[98] Malińska M., Zużewicz K., Bugajska J., Grabowski A. (2015). Heart rate variability (HRV) during virtual reality immersion. Int. J. Occup. Saf. Ergon..

[99] Gavgani A.M., Hodgson D.M., Nalivaiko E. (2017). Effects of visual flow direction on signs and symptoms of cybersickness. PLoS ONE.

[100] Park S., Ha J., Kim L. (2022). Effect of Visually Induced Motion Sickness from Head-Mounted Display on Cardiac Activity. Sensors.

[101] Hsin L.J., Chao Y.P., Chuang H.H., Kuo T.B.J., Yang C.C.H., Huang C.G., Kang C.J., Lin W.N., Fang T.J., Li H.Y. (2023). Mild simulator sickness can alter heart rate variability, mental workload, and learning outcomes in a 360° virtual reality application for medical education: A post hoc analysis of a randomized controlled trial. Virtual Real..

[102] Yang A.H.X., Kasabov N.K., Cakmak Y.O. (2023). Prediction and detection of virtual reality induced cybersickness: A spiking neural network approach using spatiotemporal EEG brain data and heart rate variability. Brain Inf..

[103] Sameri J., Coenegracht H., Van Damme S., De Turck F., Vega M.T. (2024). Physiology-driven cybersickness detection in virtual reality: A machine learning and explainable AI approach. Virtual Real..

[104] Tian N., Boulic R. (2024). Who says you are so sick? An investigation on individual susceptibility to cybersickness triggers using EEG, EGG and ECG. IEEE Trans. Vis. Comput. Graph..

[105] Choi M.H., Kang K.Y., Lee T.H., Choi J.S. (2024). Correlations between SSQ Scores and ECG Data during Virtual Reality Walking by Display Type. Appl. Sci..

[106] Wang J.Q., Qi R.R., Pan L.L., Zhou W., Zhang L.L., Cai Y.L. (2016). Motion Sickness and Resting Energy Expenditure in Chinese Male Adults. Aerosp. Med. Hum. Perform..

[107] Irmak T., Pool D.M., Happee R. (2021). Objective and subjective responses to motion sickness: The group and the individual. Exp. Brain Res..

[108] Henry E.H., Bougard C., Bourdin C., Bringoux L. (2023). Car sickness in real driving conditions: Effect of lateral acceleration and predictability reflected by physiological changes. Transp. Res. Part F Traffic Psychol. Behav..

[109] Schneider E.N., Buchheit B., Flotho P., Bhamborae M.J., Corona-Strauss F.I., Dauth F., Alayan M., Strauss D.J. (2022). Electrodermal Responses to Driving Maneuvers in a Motion Sickness Inducing Real-World Driving Scenario. IEEE Trans. Hum. Mach. Syst..

[110] Karjanto J., Yusof N.M., Terken J., Delbressine F., Rauterberg M. (2022). Level of motion sickness based on heart rate variability when reading inside a fully automated vehicle. Mech. Eng. Soc. Ind..

[111] Munoz M.L., van Roon A., Riese H., Thio C., Oostenbroek E., Westrik I., de Geus E.J.C., Gansevoort R., Lefrandt J., Nolte I.M. (2015). Validity of (Ultra-)Short Recordings for Heart Rate Variability Measurements. PLoS ONE.

[112] Melo H.M., Martins T.C., Nascimento L.M., Hoeller A.A., Walz R., Takase E. (2019). Ultra-short heart rate variability recording reliability: The effect of controlled paced breathing. Ann. Noninvasive Electrocardiol..

[113] Goldstein D.S., Bentho O., Park M.Y., Sharabi Y. (2011). Low-frequency power of heart rate variability is not a measure of cardiac sympathetic tone but may be a measure of modulation of cardiac autonomic outflows by baroreflexes. Exp. Physiol..

[114] Francesco B., Grazia B.M., Emanuele G., Valentina F., Sara C., Chiara F., Riccardo M., Francesco F. (2012). Linear and Nonlinear Heart Rate Variability Indexes in Clinical Practice. Comput. Math. Methods Med..

[115] Latchman P.L., Gates G., Pereira J., Axtell R R., Gardner K., Schlie J., Yang Q., Yue T., Morin-Viall A., DeMeersman R. (2020). The association between sympatho-vagal balance and central blood pressures. Physiol. Int..

[116] Goldberger J.J., Ahmed M.W., Parker M.A., Kadish A.H. (1994). Dissociation of heart rate variability from parasympathetic tone. Am. J. Physiol..

[117] Jacob D., Aubonnet R., Recenti M., Audardottir S.A., Ivarsdottir T.I., Burgunder B., I Escalona I.M., Colacino A., Bjornsdottir A., Petersen H. (2022). Assessing Early-stage Parkinson’s Disease Using BioVRSea. Proceedings of the 2022 IEEE International Conference on Metrology for Extended Reality, Artificial Intelligence and Neural Engineering (MetroXRAINE), Rome, Italy, 26–28 October 2022.

[118] Hell S., Argyriou V. (2018). Machine Learning Architectures to Predict Motion Sickness Using a Virtual Reality Rollercoaster Simulation Tool. Proceedings of the 2018 IEEE International Conference on Artificial Intelligence and Virtual Reality (AIVR), Taichung, Taiwan, 10–12 December 2018.

[119] Padmanaban N., Ruban T., Sitzmann V., Norcia A.M., Wetzstein G. (2018). Towards a Machine-Learning Approach for Sickness Prediction in 360° Stereoscopic Videos. IEEE Trans. Vis. Comput. Graph..

[120] Lee T.M., Yoon J.-C., Lee I.-K. (2019). Motion Sickness Prediction in Stereoscopic Videos using 3D Convolutional Neural Networks. IEEE Trans. Vis. Comput. Graph..

[121] Li Y., Liu A., Ding L. (2019). Machine learning assessment of visually induced motion sickness levels based on multiple biosignals. Biomed. Signal Process. Control.

[122] Ruiz Colmenares J.A., Asua Uriarte E., Del Campo I. (2023). Driving-Style Assessment from a Motion Sickness Perspective Based on Machine Learning Techniques. Appl. Sci..

[123] Recenti M., Jacob D., Aubonnet R., Burgunder B., I Escalona I.M., Gunnarsson A.E., Ciliberti F.K., Forni R., Donisi L., Petersen H. (2022). Predicting lifestyle using BioVRSea multi-biometric paradigms. Proceedings of the 2022 IEEE International Conference on Metrology for Extended Reality, Artificial Intelligence and Neural Engineering (MetroXRAINE), Rome, Italy, 26–28 October 2022.

[124] Cömert Z., Kocamaz A.F. (2017). Comparison of Machine Learning Techniques for Fetal Heart Rate Classification. Acta Phys. Pol. A.

[125] Zhao Z., Deng Y., Zhang Y., Zhang Y., Zhang X., Shao L. (2019). DeepFHR: Intelligent prediction of fetal Acidemia using fetal heart rate signals based on convolutional neural network. BMC Med. Inform. Decis. Mak..

[126] Ricciardi C., Improta G., Amato F., Cesarelli G., Romano M. (2020). Classifying the type of delivery from cardiotocographic signals: A machine learning approach. Comput. Methods Programs Biomed..

[127] Ricciardi C., Ponsiglione A.M., Scala A., Borrelli A., Misasi M., Romano G., Russo G., Triassi M., Improta G. (2022). Machine Learning and Regression Analysis to Model the Length of Hospital Stay in Patients with Femur Fracture. Bioengineering.

[128] Ricciardi C., Amato F., Tedesco A., Dragone D., Cosentino C., Ponsiglione A.M., Romano M. (2023). Detection of Suspicious Cardiotocographic Recordings by Means of a Machine Learning Classifier. Bioengineering.

[129] Ai Q., Liu Z., Meng W., Liu Q., Xie S.Q. (2023). Machine Learning in Robot-Assisted Upper Limb Rehabilitation: A Focused Review. IEEE Trans. Cogn. Dev. Syst..

[130] Cesarelli G., Donisi L., Amato F., Romano M., Cesarelli M., D’Addio G., Ponsiglione A.M., Ricciardi C. (2023). Using Features Extracted From Upper Limb Reaching Tasks to Detect Parkinson’s Disease by Means of Machine Learning Models. IEEE Trans. Neural Syst. Rehabil. Eng..

